# Accessible gene borders establish a core structural unit for chromatin architecture in *Arabidopsis*

**DOI:** 10.1093/nar/gkad710

**Published:** 2023-09-01

**Authors:** Hongwoo Lee, Pil Joon Seo

**Affiliations:** Department of Chemistry, Seoul National University, Seoul 08826, Korea; Department of Chemistry, Seoul National University, Seoul 08826, Korea; Plant Genomics and Breeding Institute, Seoul National University, Seoul 08826, Korea

## Abstract

Three-dimensional (3D) chromatin structure is linked to transcriptional regulation in multicellular eukaryotes including plants. Taking advantage of high-resolution Hi-C (high-throughput chromatin conformation capture), we detected a small structural unit with 3D chromatin architecture in the *Arabidopsis* genome, which lacks topologically associating domains, and also in the genomes of tomato, maize, and *Marchantia polymorpha*. The 3D folding domain unit was usually established around an individual gene and was dependent on chromatin accessibility at the transcription start site (TSS) and transcription end site (TES). We also observed larger contact domains containing two or more neighboring genes, which were dependent on accessible border regions. Binding of transcription factors to accessible TSS/TES regions formed these gene domains. We successfully simulated these Hi-C contact maps via computational modeling using chromatin accessibility as input. Our results demonstrate that gene domains establish basic 3D chromatin architecture units that likely contribute to higher-order 3D genome folding in plants.

## Graphical Abstract

**Graphical Abstract:**
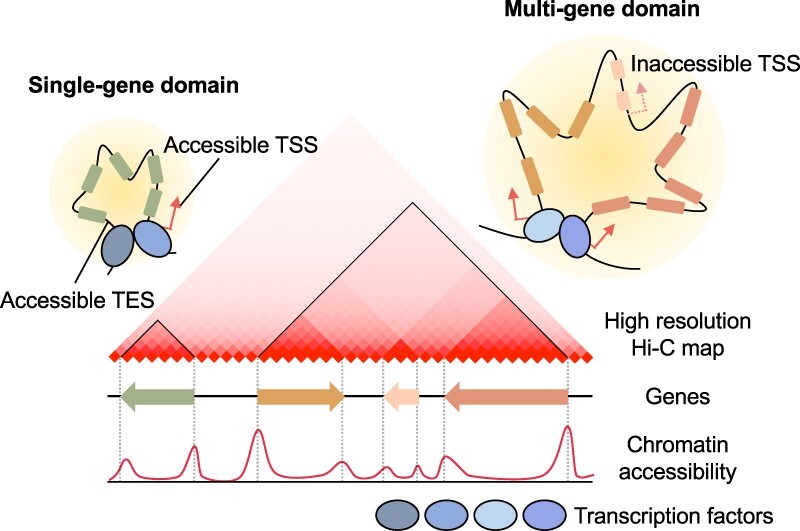

## INTRODUCTION

Recent developments in genome-wide sequencing techniques, such as Hi-C (high-throughput chromatin conformation capture) and its variant Micro-C, have allowed the elucidation of general principles behind the three-dimensional (3D) chromatin folding of eukaryotic genomes (1,2). Genome folding generally involves hierarchical structures ranging from chromatin loops to chromosome territories (3). The best-known 3D chromatin organization units are topologically associating domains (TADs), which show a self-interacting pattern with strongly interacting boundaries in Hi-C contact maps of eukaryotic genomes (4,5). Genome architectural proteins, such as CTCF (CCCTC-binding factor) and cohesin, bind strongly to DNA anchor sites and mediate the formation of chromatin contact domains through loop extrusion (6–10). In addition to TADs, structural units called compartmental domains have been demonstrated in animals (11–13). Compartmental domains exhibit both intra-domain and inter-domain interactions that are independent of CTCF and cohesin (11,14). Instead, these domains are closely associated with local chromatin states. Compartmental domains preferentially interact with other compartmental domains of similar chromatin states, contributing to the establishment of the 3D architecture for a given genome (11–13).

Plant genomes also frequently exhibit a higher-order 3D chromatin organization. TADs or TAD-like structures have been described in tomato (*Solanum lycopersicum*), maize (*Zea mays*), rice (*Oryza sativa*), sorghum (*Sorghum bicolor*), foxtail millet (*Setaria italica*), upland cotton (*Gossypium hirsutum*) and pepper (*Capsicum annuum*) (15–20), although their genomes do not encode CTCF homologs (21). Notably, plant TAD-like structures share several features with compartmental domains, including a lack of strong interactions at domain boundary regions and a dependence on chromatin state (12,15). In addition, other types of plant-specific contact domains have been described (17,22,23). For instance, intergenic condensed spacer (ICONS) structures that are similar to TADs are present in hexaploid wheat (*Triticum aestivum*), in which genes are enriched at ICONS borders but depleted within ICONS (22). Similarly, TAD-like domains in the pepper genome form as gene-to-gene loops (17). Moreover, TCP1 (TEOSINTE BRANCHED 1, CYCLOIDEA and PCF1)-rich TADs have been identified in the nonvascular plant *Marchantia polymorpha* (*M. polymorpha*) (23), and plant-specific TCP1 binding sites are enriched at boundary regions (23). Although plant-specific 3D chromatin domains have been identified in various species, general principles and mechanisms underlying their formation remain elusive.

In contrast to other plant species, *Arabidopsis* (*Arabidopsis thaliana*) lacks plant-type TADs (24–26). The absence of TADs in the *Arabidopsis* genome is likely related to its small size (∼135 Mb), high gene density (1 gene per 4.35 kb), and short intergenic regions (27). Despite the lack of obvious 3D interacting domains in *Arabidopsis* Hi-C contact maps, a hidden Markov model-based approach has revealed >1000 chromatin regions that exhibit TAD boundary-like characteristics such as DNase I hypersensitivity (28). Moreover, many chromatin loops, especially gene loops that are formed within a single-gene unit, have also been identified in *Arabidopsis* (29–32). Additionally, HiChIP and Capture-Hi-C (C-Hi-C) analyses suggest that compartmental domains are present in *Arabidopsis* (33). For instance, Polycomb-associated repressive domains enriched with trimethylation of lysine 27 from histone H3 (H3K27me3) establish compartmental domains and interact with each other (33). These lines of evidence indicate that 3D chromatin organization might exist in the *Arabidopsis* genome, but our understanding of the underlying chromatin folding mechanism is limited.

In this study, we present an integrative genome-wide analysis of 3D chromatin folding patterns using high-resolution Hi-C data of the *Arabidopsis* genome. We discovered that a single gene or multiple neighboring genes can form domain-like structures (hereafter referred to as gene domains) that are conserved across several plant species. Binding of transcription factors to accessible transcription start sites (TSSs) and transcription end sites (TESs) drives the formation of basic structural units of chromatin, contributing to global 3D genome architectures in plants.

## MATERIALS AND METHODS

### Hi-C data processing

Raw sequencing reads were downloaded from NCBI Sequence Read Archive (SRA; http://www.ncbi.nlm.nih.gov/sra/;Supplementary Table S1). Sequencing files of plant species were processed by Juicer (v1.22.01) (34) with the reference genomes of *Arabidopsis thaliana* (TAIR10), tomato (SL4.0), maize inbred line B73 (NAM5.0), and *Marchantiapolymorpha* (Tak1v5.1). Mapped reads were filtered with a mapping quality cutoff value of 30. Normalized or nonnormalized contact matrices at single- or two-fragment resolution were extracted from .hic files using the Dump command in Juicer Tools (34) (Supplementary Table S2). KR normalization was applied to *Arabidopsis*, tomato and maize data, whereas square root vanilla coverage (VC_SQRT) normalization was applied to *M. polymorpha* data. Normalized Hi-C contact maps were visualized using JuiceBox (34).

### DI score calculation

DI scores were calculated as previously described (4). In *Arabidopsis* and *M. polymorpha*, normalized observed/expected (OE) values of Hi-C reads at single-restriction-fragment resolution were used to calculate DI scores. For tomato and maize genome analysis, those at two-restriction-fragment resolution were used for DI calculation due to the relatively low resolution of Hi-C raw data. Contacts with other restriction fragments within 10 kb regions upstream or downstream of each fragment were added to calculate *A* (upstream contact) or *B* (downstream contact). *E* was calculated as (*A* + *B*)/2.


\begin{equation*}{\mathrm{DI}} = \left( {\frac{{B - A}}{{\left| {B - A} \right|}}} \right) \times \left( {{\mathrm{\ }}\frac{{{{\left( {A - E} \right)}}^2}}{E} + \frac{{{{\left( {B - E} \right)}}^2}}{E}} \right).\end{equation*}

### Metagene analysis

Metagene profiles of DI scores, the first eigenvector of Pearson's correlation Hi-C matrix (first eigenvector) and epigenetic marks were obtained from bigWig files using deepTools (v3.5.0) (35). The bigWig files of DI scores were processed using in-house scripts, and those of epigenetic marks were downloaded from Plant Chromatin State Database (PCSD; http://systemsbiology.cau.edu.cn/chromstates) (36). The raw sequencing reads of H2A and H3 chromatin immunoprecipitation-sequencing (ChIP-seq) were downloaded from SRA database and processed to bigWig files (Supplementary Table S1). *Arabidopsis* protein-coding genes (*n* = 27 445), except for the genes at blacklisted regions (37) (*n* = 117) having systematically high signal, were used in the metagene analysis.

### Pile-up analysis

To visualize high-resolution (single- or two-restriction-fragment resolution) contact maps, we piled-up the images of a large number of genes (*n* > 100) to avoid bias caused by restriction cut sites and noise at a gene scale. We used OE values for pile-up analysis instead of conventional observed values, because gene domain structures usually did not have strong contacts at anchoring sites, unlike canonical TADs. Genic and surrounding regions of individual genes were used for pile-up analysis. To preserve the image ratio, the length of the surrounding regions (padding: 0.5 × gene length or 1 × gene length) was determined by the length of each gene. Normalized OE values were initially assigned to the *L* × *L* matrix (where *L* = gene length + 2 × padding). To pile up images of individual genes, the *L* × *L* matrix was resized to an 80 × 80 matrix using bicubic interpolation. An average matrix was calculated and visualized as a heatmap. For single-gene domain pile-up analysis, (–) stranded genes were flipped to correct for orientation. In multigene domain pile-up analysis, gene pairs with an intergenic distance of 40 kb or more were filtered out. Surrounding regions within a distance of 0.5× multigene domain length were included for pile-up analysis. Protein-coding genes of *Arabidopsis* and *M. polymorpha* were used in the pile-up analysis. In tomato and maize, genes shorter than 50 kb were used for the pile-up analysis.

### RNA-sequencing analysis

RNA-sequencing reads used in this study were downloaded from SRA database (Supplementary Table S1). Reads were mapped using STAR (v2.7.10a) with the parameters ‘-pOverlapNbasesMin 12 -peOverlapMMp 0.1 -twopassMode Base’ (38). RSEM (v1.3.1) was employed to estimate transcript abundance (39), and differentially expressed genes were identified by Deseq2 (v1.34.0) (40) with a threshold of log_2_(fold change) > 1 and *P* value < 0.05 (Supplementary Table S3).

### Assay for transposase-accessible chromatin using sequencing (ATAC-seq) and ChIP-seq data analysis

The bigWig file of ATAC-seq data from *Arabidopsis* was downloaded from PCSD (36). The bigWig file was converted to bedGraph to calculate chromatin accessibility at TSSs and TESs (41). For quantification of chromatin accessibility at TSSs or TESs, the maximum value of chromatin accessibility within 100 bp on either side of TSSs or TESs was measured. For ATAC-seq analysis of tomato, maize and *M. polymorpha*, we downloaded .fastq files from SRA database (Supplementary Table S1). Reads were trimmed using Trim-galore (v0.6.7) (10.5281/zenodo.5127899) before being mapped to the corresponding genome using Bowtie2 (v2.4.5) (42) with default parameters. Bins per million (BPMs) were calculated with a bin size of 10 bp using bamCoverage (35). Chromatin accessibility at TSSs or TESs was measured as the maximum value of chromatin accessibility within 100 bp on either side of TSSs or TESs.

For transcription factor ChIP-seq analysis, processed ChIP-seq data were downloaded from previous reports (23,43). For ChIP-seq analysis of chromatin remodelers, raw read files for BRM and CHR11 of *Arabidopsis* were downloaded (44,45). Downloaded reads were trimmed and mapped to the *Arabidopsis* TAIR10 as in the ATAC-seq analysis. MACS2 (v2.2.7.1) (46) was employed to call DNA-binding peaks. Genes having ChIP-seq peaks of transcription factors and chromatin remodelers from 500 bp upstream of the TSS to the TES were annotated as target genes. Common target genes in all biological replicates were used for the pile-up analysis (Supplementary Table S4 and S5).

### Correlation analysis between subregional chromatin contacts and chromatin features

To identify specific chromatin features associated with subregional chromatin contacts, enrichment levels of each chromatin feature at TSS, TES and gene body regions were measured for the correlation analysis. Chromatin feature enrichment at TSS and TES was estimated using the maximum ChIP-enrichment level of each chromatin feature within 100 bp on either side of TSS and TES, whereas chromatin feature enrichment at gene body region was obtained from an average value of ChIP-enrichment levels within a gene body region. Subsequently, we calculated the Spearman's correlation coefficient between the sum of chromatin contacts within each subregion of Hi-C contact image (G and S-A to S-E regions) and the enrichment level of each chromatin feature at TSS, TES or gene body.

### Gene domain identification

To identify chromatin accessibility-dependent gene domains, chromatin accessibility levels at TSS and TES were measured as described in the ATAC-seq analysis. To profile gene domain structure, genes longer than 2 kb were divided into 10 quantiles (Q1–Q10) according to the values of TSS accessibility multiplied by those of TES accessibility (TSS accessibility × TES accessibility). For multigene domain identification, the values of accessibility at the left border of the first gene multiplied by those at the right border of the last gene (LB accessibility × RB accessibility) were used for dividing 10 multigene domain quantiles. Hi-C images of genes in each quantile were piled-up and visualized to examine the formation of gene domains. The strength of gene domain structure (Domain Score, DS) was calculated as the ratio of mean OE values in G subregion to mean OE values in S-C subregion of Hi-C contact map.

### DAP-seq data analysis

For the *Arabidopsis* DAP-seq analysis, processed DAP-seq peak data for 470 transcription factors were downloaded from NCBI (47). As for the maize DAP-seq analysis, raw sequencing reads were downloaded from SRA database and processed as described in the ATAC-seq analysis. DAP-seq peaks were called using GEM (v3.4) (48) with the GST-HALO negative control sample as previously reported (49). Peak calling was performed using the parameters of ‘–d Read_Distribution_default.txt –k_min 6 –k_max 20’, and a cut-off value of FDR $ \le$ 0.01 was applied. DAP-seq peaks located around the TSS or TES region of each gene (100 bp on either side of the TSS or TES) were used for downstream analysis.

### Compartment analysis

To define A/B compartments, we obtained the Pearson's correlation matrix of normalized Hi-C contact maps at both 5-restriction-fragment and 50-restriction-fragment resolution using Juicer Tools (34). The first eigenvector of the correlation matrix was calculated using numpy.linalg.eig (50). Then, A/B compartments were assigned to each 5- or 50-restriction-fragment bin based on the sign (+/$ -$) of the first eigenvector value. To explore A/B compartments in the genic regions, we utilized BEDTools intersect command (51). All *Arabidopsis* genes were annotated as A, B, AB (gene with a transition from an A compartment to a B compartment in a 5′ → 3′ direction in its genic region), BA (gene with a transition from a B compartment to an A compartment in a 5′ → 3′ direction in its genic region), ABA, or BAB compartment at 5-restriction-fragment and 50-restriction-fragment resolution. Genes with more complex compositions were excluded from the analysis. To examine association between chromatin features and local A/B compartments, each 5-restriction-fragment bin was annotated as A (first eigenvector value >0.002; the number of bins = 22 160), B (first eigenvector value <−0.003; the number of bins = 21 932) or none (the number of bins = 20 251), based on the first eigenvector value.

### Hi-C data simulation

The chromatin accessibility levels at TSSs and TESs were used as inputs to the simulation model. Chromatin accessibility at TSSs and TESs was calculated as described in the ATAC-seq analysis. In addition, information on positions of TSSs and TESs, and the orientation of genes were used for searching for potential anchoring sites.

The model was generated with two terms, a backbone distance-decay term and an accessibility-dependent contact term. As the distance-decay model is known to follow power-law decay, we designed the backbone model (${\mathrm{M}}$) with two constants (*a*1 and *b*1) and a distance between two bins at two-restriction-fragment resolution (*j*$ -$*i*) as shown below:


\begin{equation*}{\mathrm{M}}\left( {i,j} \right) = a1 \times {\left( {j - i} \right)}^{b1}.\end{equation*}

We added the accessibility-dependent contact term with another distance-decay model. To identify accessibility-dependent interacting TSSs and TESs, we utilized two additional hyperparameters, the maximum distance between anchors and minimum accessibility values at anchoring sites. We also considered the directionality of chromatin contacts at TSS and TES, which interacts toward downstream and upstream chromatin regions, respectively. The maximum distance was set to 100 restriction fragments, and the minimum accessibility value was set to 1.0. For each identified interacting pair $( {{x}_i,{x}_j} )$, we added accessibility-dependent contacts for every element $( {{x}_k,{x}_l} )$$( {{\mathrm{k}},{\mathrm{l}} = {\mathrm{i}},{\mathrm{\ i}} + 1 \ldots {\mathrm{j}}} )$ between the interacting regions. *AC_i_* and *AC_j_* indicate chromatin accessibility values at the interacting pair. The accessibility-dependent contact $( {\mathrm{C}} )$ for an interacting pair $( {{x}_i,{x}_j} )$ was calculated as shown below:


\begin{equation*}{\mathrm{C}}\left( {{{\mathrm{x}}}_k,{{\mathrm{x}}}_l} \right) = \left( {A{C}_i + A{C}_j} \right) \times a2 \times {\left( {{{\mathrm{x}}}_l - {{\mathrm{x}}}_k} \right)}^{b2},\end{equation*}

\begin{equation*}\left( {{x}_i \le {{\mathrm{x}}}_k < {{\mathrm{x}}}_l \le {\mathrm{\ }}{x}_j} \right). \end{equation*}

The accessibility-dependent contact $( {\mathrm{C}} )$ was added to the backbone contact $( {\mathrm{M}} )$, and a Poisson distribution was applied to a simulated matrix to mimic noise to each bin.

To determine hyperparameters *a*1, *b*1, *a*2 and *b*2, we conducted simulations of OE maps for every 300-restriction-fragment segment within the 1–5 245 117 bp regions of chromosome 1. The average root mean squared error (RMSE) and Spearman's correlation coefficient were calculated after applying a wide range of hyperparameters (Supplementary Table S6). Since we observed a trade-off between Spearman's correlation coefficient and RMSE, the combinations of hyperparameters that maximized the Spearman's correlation coefficient while maintaining an RMSE cutoff of 1.28 were chosen (Supplementary Table S6). Ultimately, we obtained the hyperparameter values of *a*1 = 160, *b*1 = −1.3, *a*2 = 0.2, *b*2 = −0.5 for our simulation model. Hyperparameters of *a*1 = 160, *b*1 = −1.3, *a*2 = 0, *b*2 = 0 were used for the control simulation.

### Conventional TAD analysis

To investigate overlaps between gene domain and TAD boundaries in various plant genomes, we called TADs in tomato, maize and *M. polymorpha* genomes using HiTAD algorithm (52) that has been widely used for the identification of TADs in large-genome plants (15). We called TADs with the parameters of ‘-W KR –maxsize 200000’ at 5 kb resolution. TAD boundaries defined at a 5 kb resolution were compared with gene domain boundaries. To identify gene domain boundaries, TSSs and TESs were divided each into 10 quantiles based on the chromatin accessibility level, and gene domain boundaries of Q1 (strong gene domain boundaries) and Q10 (weak gene domain boundaries) were collected. For control analysis (shifted control), 10 kb upstream regions of TSSs or 10 kb downstream regions of TESs were collected. BEDTools (51) was employed to examine overlap between TAD and gene domain boundaries.

### Data visualization

All heatmaps and 1D plots were generated with matplotlib (53) and seaborn (54).

## RESULTS

### High-resolution hi-C analysis reveals conserved single-gene domains in plant genomes

**Figure 1.**
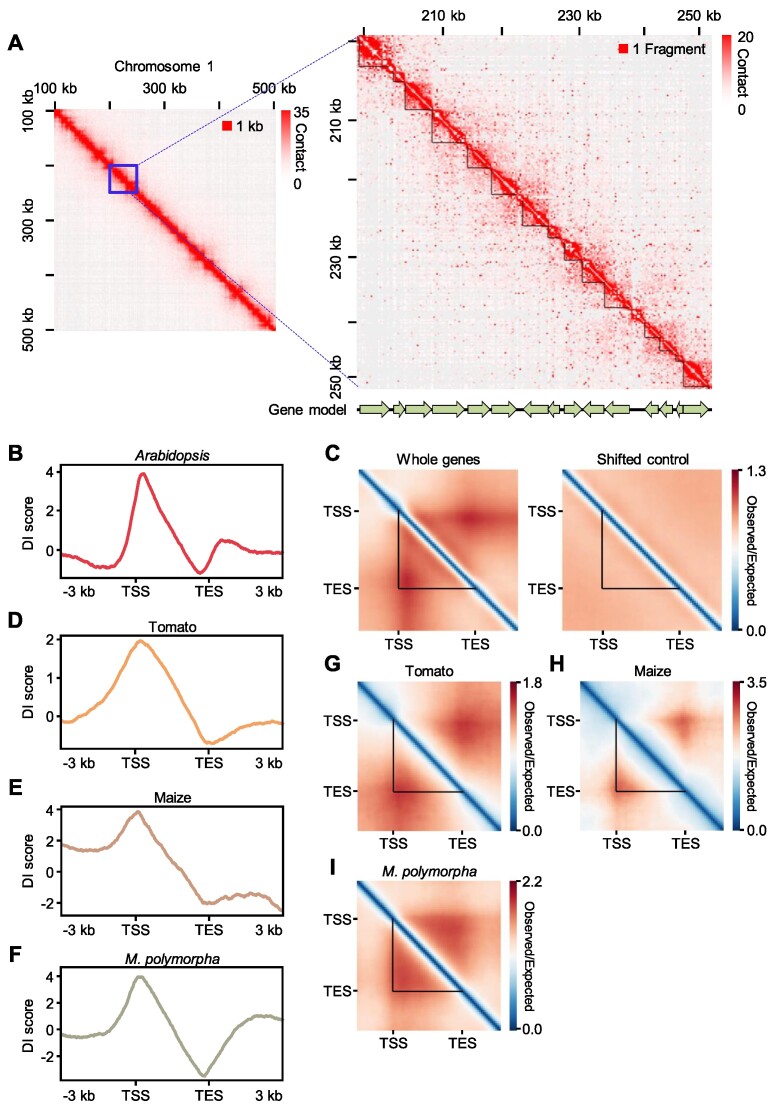
Single-gene domains are conserved in plants. (**A**) KR-normalized Hi-C contact heatmaps at 1 kb resolution (left) and single-restriction-fragment resolution (right). (**B**) Metaplot of DI scores for all individual *Arabidopsis* genes. (**C**) Pile-up images of Hi-C contact matrices of all individual *Arabidopsis* genes (left panel). The result of same analysis is shown for the 5 kb upstream regions of all individual genes as a control (shifted control, right panel). (D–F) Metaplots of DI scores for tomato (**D**), maize (**E**) and *M. polymorpha* (**F**) genes. (G–I) Pile-up images of Hi-C contact matrices of tomato (**G**), maize (**H**) and *M. polymorpha* (**I**) genes. In (G) and (H), genes longer than 3 kb were included in the analysis. In (I), genes longer than 2 kb were included in the analysis. In (A), (C) and (G–I), the black triangles indicate gene boundaries.

Although the presence and extent of 3D chromatin architecture in the *Arabidopsis* genome have not been proven, growing evidence suggests that significant 3D chromatin structures may be present. Considering the small size of the *Arabidopsis* genome, a high-resolution Hi-C analysis is necessary to identify a basic chromatin domain present across the genome. We reanalyzed the most-deeply sequenced available Hi-C dataset (Supplementary Table S2) of the *Arabidopsis* genome (55) and constructed a Hi-C map at single-restriction-fragment resolution, which is the highest resolution that can be theoretically achieved in this type of experiment (2). We normalized the raw Hi-C matrix according to the Knight–Ruiz (KR) matrix-balancing method to correct for sequencing biases (56,57). At this single-restriction-fragment resolution, we detected self-interacting domains from the Hi-C contact map whose boundaries closely matched gene boundaries (Figure 1A), similar to gene mini-domains identified in *Drosophila melanogaster* (12) (see Discussion). We explored whether these gene domains existed globally by calculating the directionality index (DI) score, which represents the directionality of chromatin contacts (4), for all individual *Arabidopsis* genes. After normalization of the distance between two interacting regions (observed/expected [OE] values of a paired end Hi-C read) (57), the genome-wide pattern of DI scores showed a peak with a high positive value over TSSs (interacting toward downstream regions), together with a peak with a negative value over TESs (interacting toward upstream regions) (Figure 1B). This result suggests that chromatin contacts are globally observed in genic regions within the boundaries of TSSs and TESs.

To better understand chromatin contact patterns around genic regions, we performed a pile-up analysis for genic and surrounding regions of all individual *Arabidopsis* genes using OE values as a measurement of chromatin contacts. Individual genes formed domain-like structures with interaction over the entire gene body (self-interaction) insulated between the TSS and TES (hereafter called single-gene domains) (Figure 1C), regardless of matrix normalization methods, padding sizes, and datasets (Supplementary Figure S1). As a control, we also performed a pile-up analysis using 5 kb sequences upstream of all individual genes (shifted control) and observed no distinct interaction patterns (Figure 1C). These results suggest that individual genes frequently form single-gene domains flanked by the TSS and TES in *Arabidopsis*.

We investigated whether these single-gene domains also existed in other plant species. We analyzed Hi-C data from tomato (15), maize (15), and *M. polymorpha* (23) at single- or two-restriction-fragment resolution. Although sequencing depth and distance-decay models of these Hi-C datasets were different (Supplementary Table S2; Supplementary Figure S2), we observed genome-wide patterns of DI scores within genic regions that were similar to those seen in *Arabidopsis* (Figure 1D–F). Pile-up analyses also showed that chromatin contacts were particularly enriched in gene body regions within the boundaries of the TSSs and TESs (Figure 1G–I), indicating that single-gene domains are conserved in these plant species.

### Single-gene domain structures are associated with gene expression

**Figure 2.**
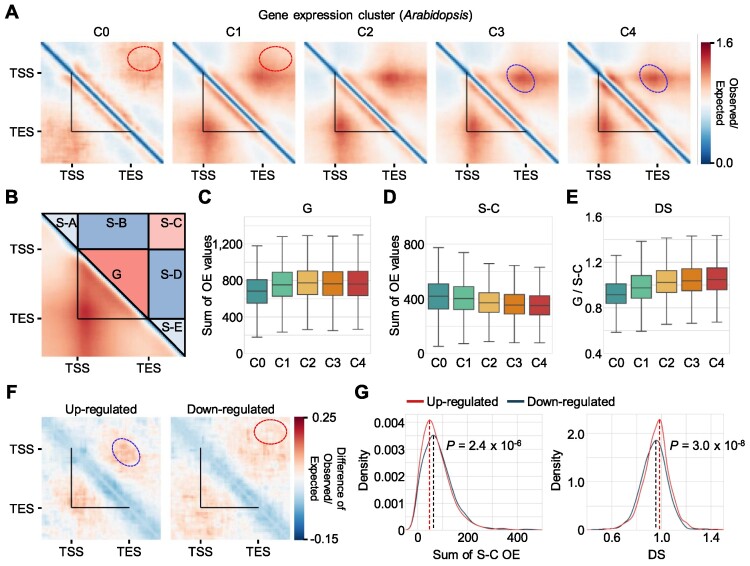
Single-gene domains are associated with transcriptional states. (**A**) Pile-up images of Hi-C contact matrices of *Arabidopsis* genes clustered by gene expression levels. Genes longer than 2 kb (*n* = 14 411) were divided into five clusters according to their expression levels from C0 (not expressed) to C4 (highly expressed). (**B**) Diagram of subregions in Hi-C contact matrices, based on gene borders (G, gene body; S-A to S-E, surrounding regions A–E). (**C**) Sum of chromatin contacts (observed/expected [OE] values of Hi-C reads) within the G subregion in each expression quantile of *Arabidopsis* genes. (**D**) Sum of OE values within the S-C subregion in each expression quantile of *Arabidopsis* genes. (**E**) Domain score (DS) in each expression quantile. DS was calculated as the mean G contact value (OE/pixel) divided by the mean S-C contact value (OE/pixel). (**F**) Structural changes of gene domains in response to heat. Heat and control *Arabidopsis* Hi-C contact maps were generated using upregulated (*n* = 2 377) and downregulated genes (*n* = 2 179), respectively, in response to heat. Difference in the pile-up images between upregulated (left panel) and downregulated (right panel) genes in response to heat is shown (heat condition − mock condition). (**G**) Kernel density plots of the S-C contact value (OE value) difference and DS difference. Hi-C contact maps shown in (F) were used for analysis. The *P* value was calculated by a two-sided Mann–Whitney test. In (A) and (F), the black triangles indicate gene boundaries. Red circles indicate S-C subregion, whereas blue circles indicate a TSS–TES contact site. In (C–E), the box represents the interquartile range of the data, and the horizontal line indicates the median value. The whiskers indicate 1.5 times the interquartile range. Outliers are not shown.

Since 3D chromatin domains are usually linked to transcriptional control (58–60), we investigated whether single-gene domains were associated with the transcriptional state of their constituent genes. We retained only *Arabidopsis* genes more than 2 kb in length (*n* = 14 411), as short genes do not usually generate the sufficient number of restriction fragments for contact site dissection in raw Hi-C datasets. We divided the selected genes into five clusters according to their expression levels, from not expressed (C0) to highly expressed (C4) (Supplementary Figure S3A). Highly expressed genes (clusters C3 and C4) showed a strong domain structure (Figure 2A, blue circles). By contrast, rarely expressed genes (clusters C0 and C1) did not have an obvious TSS–TES anchor but rather showed contact enrichment outside of gene body regions (Figure 2A, red circles). We performed the same analysis using all genes without length cutoff (*n* = 27 322) and obtained similar results, albeit with a relatively lower image resolution (Supplementary Figure S4). These results indicate that single-gene domain structures correlate with expression levels.

We noticed that chromatin contacts of genes in clusters C3 and C4 were enriched in genic regions, whereas those of genes in clusters C0 and C1 mainly formed chromatin contacts between the intergenic sequences beyond TSS and TES (Figure 2A). Thus, we partitioned each Hi-C contact image into six subregions: the gene body (G) and surrounding (S) regions S-A to S-E beyond the gene borders (Figure 2B). We calculated the sum of OE values at each subregion and looked for correlations between chromatin contacts within a particular subregion and gene expression levels (Figure 2C, D; Supplementary Figure S3). Chromatin contacts within G regions were positively correlated with gene expression levels, whereas chromatin contacts within S-C regions showed a negative correlation with gene expression levels (Figure 2C, D). According to these results, we defined a domain score (DS) as the mean OE value in the G region divided by that in the S-C region (mean OE in G/mean OE in S-C) and used this as a measure of insulation strength of gene domain relative to surroundings. DS values were related to gene expression levels (Figure 2E). We detected a similar relationship between single-gene domain structures and gene expression levels in tomato, maize and *M. polymorpha* (Supplementary Figure S5). This relationship suggests that the single-gene domain structure is associated with gene expression level and that highly expressed genes tend to form strong single-gene domain structures in plant genomes.

We assessed whether dynamic changes in gene expression accompany conformational changes in single-gene domain structures in response to internal or external stimuli. Pile-up analysis using high-resolution Hi-C data of heat-treated *Arabidopsis* seedlings (55) (Supplementary Figure S6) showed that chromatin contacts within S-C regions markedly increased for genes downregulated by heat treatment, whereas DS values increased in genes upregulated by heat treatment (Figure 2F, G; Supplementary Table S3). These results indicate that the local 3D chromatin structures at genic and surrounding regions can dynamically change to reflect the transcriptional state of its constituent gene.

### Chromatin accessibility determines the formation of single-gene domains

**Figure 3.**
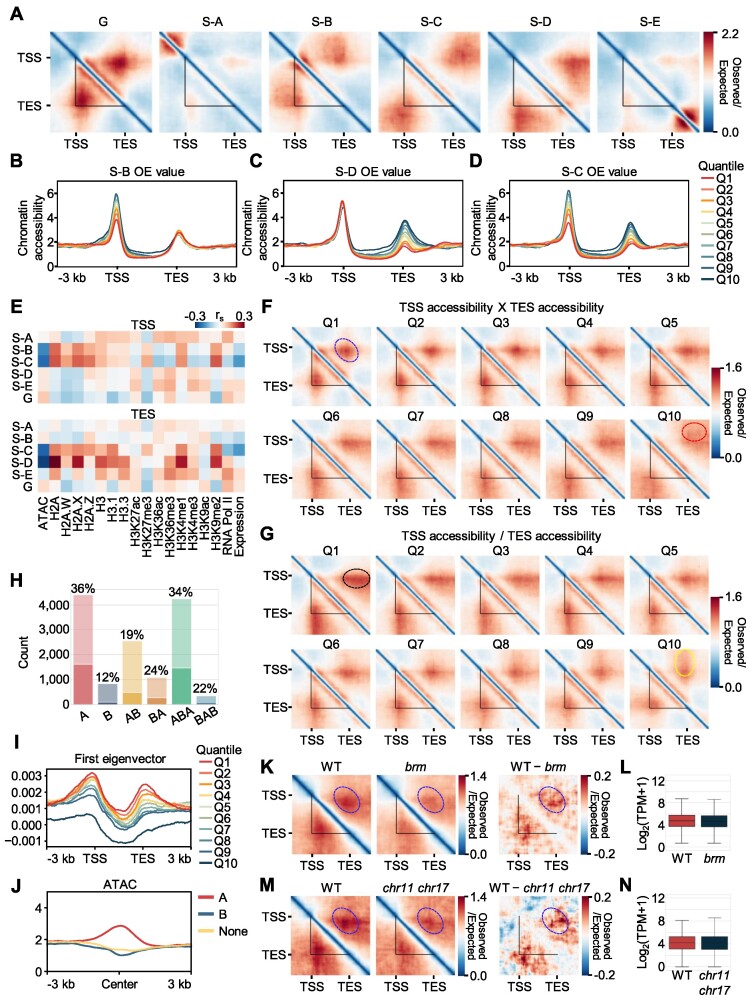
Chromatin accessibility is responsible for the formation of single-gene domains. (**A**) Pile-up images of Hi-C contact matrices of *Arabidopsis* genes with the top 10% OE values in each subregion. (B–D) Metaplots of chromatin accessibility for each OE value quantile. Accessibility levels in each OE value quantile at S-B (**B**), S-D (**C**) and S-C (**D**) regions are shown. (**E**) Correlation heatmaps between the sum of chromatin contacts at each subregion and the enrichment level of chromatin features at TSS and TES. Spearman's correlation coefficients (*r*_s_) are shown. (**F**) Domain structures of genes clustered by TSS accessibility × TES accessibility. Genes longer than 2 kb were clustered into 10 quantiles according to the values of TSS accessibility × TES accessibility. (**G**) Domain structures of genes clustered by TSS to TES accessibility ratio (TSS accessibility/TES accessibility). Genes longer than 2 kb were divided into 10 quantiles according to the ratio between TSS and TES accessibility. Black circles indicate extended contacts between TSS and TES-downstream sequence, whereas yellow circles indicate extended contacts between TSS-upstream sequence and TES. (**H**) Number of genes with strong gene domains in each local A/B compartment defined at 5-restiction-fragment resolution. Genes within Q1–Q3 quantiles clustered by TSS accessibility × TES accessibility were annotated as strong gene domains. Boxes with light colors indicate the total number of *Arabidopsis* genes contained in each local A/B compartment. The ratio of the number of genes with strong gene domains over total number of genes in each local compartment is provided. (**I**) Metaplot of the first eigenvector of the Pearson's correlation matrix of a normalized Hi-C contact map. The average values of the first eigenvector at genic regions of genes in each quantile divided by the values of TSS accessibility × TES accessibility are shown. (**J**) Metaplot of chromatin accessibility within each local compartment at genic regions. Fragments were annotated as A, B or none based on their first eigenvector values. (K, M) Comparison of gene domain structures between the wild type and the chromatin remodeler mutants *brm* and *chr11 chr17*. Pile-up images of Hi-C contact matrices of target genes of BRM (longer than 2 kb, *n* = 1 904) (**K**) and CHR11 (longer than 2 kb, *n* = 830) (**M**) chromatin remodelers are shown. Left panel, wild-type Hi-C contact map; middle panel, Hi-C contact map in the chromatin remodeler mutant; right panel, difference in Hi-C contact maps between the wild type and the chromatin remodeler mutant. (**L, N**) Expression levels of chromatin remodeler target genes in wild type and chromatin remodeler mutants. Log_2_(transcripts per million [TPM] + 1) values of BRM-target genes (longer than 2 kb, *n* = 1 904) (L) and CHR11-target genes (longer than 2 kb, *n* = 830) (N) are shown. In (F), (K) and (M), the red circles indicate S-C subregion, whereas the blue circles indicate a TSS–TES contact site. In (A), (F), (G), (K) and (M), the black triangles indicate gene boundaries.

We sought to explore which chromatin feature(s) determined 3D chromatin structures around genic regions. To this end, we divided genes over 2 kb in length into 10 quantiles according to the sum of OE values within each of their subregions in Hi-C contact image (Figure 3A). Then, we investigated enrichment patterns for epigenetic modifications in each quantile to look for potential links between subregional chromatin contacts and epigenetic features (Supplementary Table S1). Metaplot analysis revealed that chromatin accessibility signals were most strongly associated with subregional chromatin contacts. In general, chromatin accessibility signals were enriched at both TSS and TES, where chromatin contacts were mainly observed (Figure 3B–D; Supplementary Figures S7–S12). In particular, subregional chromatin contacts within S-B, S-C and S-D were linked to differential chromatin accessibility levels at TSS and TES (Figure 3B–D), while the enrichment patterns of other epigenetic features were not substantially changed depending on the division of subregions (S-B, S-C and S-D) (Supplementary Figures S9–S11). Correlation analysis also supported that chromatin contact strength within S-B, S-C and S-D subregions showed a strong negative correlation to the chromatin accessibility at TSS only, both TSS and TES, and TES only, respectively (Figure 3E). While many chromatin marks did not show a significant correlation, chromatin contacts within S-B, S-C and S-D subregions showed a positive correlation to gene body-enriched chromatin features (e.g. H2A.X, H3K4me1 and H3K9me2) (Figure 3E; Supplementary Figure S13). Considering that it was also the case of canonical H2A and H3 histones (Figure 3E; Supplementary Figure S13), those positive correlations were mainly attributable to a high nucleosome density that reduces chromatin accessibility.

Contacts within S-B subregions, which represent contacts between gene bodies and sequences upstream of TSSs, were negatively correlated with TSS accessibility (Figure 3B), whereas contacts within S-D subregions (representing contacts between gene bodies and sequences downstream of TESs) were negatively correlated with TES accessibility (Figure 3C). Furthermore, S-C contacts (representing contacts between sequences upstream of TSSs and those downstream of TESs) showed a negative correlation with both TSS and TES accessibility (Figure 3D). Based on these results, we proposed a model of accessible gene border-dependent chromatin contacts (Supplementary Figure S14). Genes with accessible gene borders may form distinct gene domains. However, genes with inaccessible TSS or TES likely interacts with adjacent upstream or downstream accessible regions, leading to chromatin contacts between TES and upstream regions (S-B region in Hi-C contact map) or TSS and downstream regions (S-D region in Hi-C contact map). When both TSS and TES are inaccessible, gene domains are not formed, and instead, chromatin contacts may occur between accessible chromatin regions outside of TSS and TES (S-C region in Hi-C contact map) (Supplementary Figure S14). Taken together, our results indicate that accessible TSSs and TESs serve as anchors for the formation of single-gene domains.

To further test this finding, we calculated TSS and TES accessibility for all individual genes over 2 kb in length and divided them into 10 quantiles according to the values of TSS accessibility multiplied by those of TES accessibility (TSS accessibility × TES accessibility) (Supplementary Figure S15A). We determined that genes with high accessibility at both TSSs and TESs had a strong tendency to form single-gene domains (Figure 3F, a blue circle), whereas genes with inaccessible TSSs and TESs seldom formed single-gene domains (Figure 3F, a red circle). We obtained similar results with the tomato, maize and *M. polymorpha* genomes, showing that chromatin accessibility is crucial for single-gene domain formation in various plant species (Supplementary Figure S16).

We clustered *Arabidopsis* genes into 10 quantiles according to the ratio between their TSS accessibility and TES accessibility (TSS accessibility/TES accessibility) (Supplementary Figure S15B). Genes with high TSS to TES accessibility ratios showed strong contacts between TSSs and regions downstream of inaccessible TESs (Figure 3G, a black circle). By contrast, genes with low TSS accessibility but high TES accessibility showed strong contacts between TESs and regions upstream of inaccessible TSSs (Figure 3G, a yellow circle). These results indicate that accessible TSSs or TESs participate in chromatin contacts in a directional manner; TSSs mainly interact with accessible downstream sequences, whereas TESs interact with accessible upstream sequences.

The association between chromatin contact and accessibility was independent of restriction enzyme efficiency (Supplementary Figure S17) and linear distances to neighboring genes (Supplementary Figure S18). In addition, since chromatin accessibility was closely linked to gene expression, we also wanted to determine whether chromatin accessibility is epistatic to gene expression level in gene domain formation. Notably, even rarely expressed genes (C0 and C1 of gene expression clusters; Figure 2A) formed strong gene domain structures, when their chromatin accessibility was high (Q1 and Q2 in TSS accessibility × TES accessibility quantiles; Figure 3F) (Supplementary Figure S19), indicating that chromatin accessibility is more important for gene domain formation than transcriptional activity.

We also explored whether gene domain structures are related to the A/B compartment. We first defined A/B compartments at the 50-restriction-fragment resolution with an average bin size of 13 738 bp. At this resolution, strong gene domains (Q1–Q3 of TSS accessibility × TES accessibility quantiles; Figure 3F) were found in both A and B compartments with a similar ratio (Supplementary Figure S20). We further defined the ‘local’ A/B compartment at 5-restriction fragment resolution (here after referred to as local A/B compartment). This high-resolution compartment analysis allowed the identification of local A/B compartments even within a gene. Strong gene domains were predominantly found in local A as well as local ABA compartments (Figure 3H). The first eigenvector of Pearson's correlation matrix at 5-restriction-fragment resolution exhibited peaks at TSS and TES (Figure 3I), and consistently, the local A compartment strongly correlated to chromatin accessibility at genic regions, unlike the other chromatin features (Figure 3J; Supplementary Figure S21). These results indicate that accessible gene borders have distinct local compartments at high resolution.

Because chromatin accessibility is mainly regulated by ATP-dependent chromatin remodelers (61–63), we postulated that single-gene domain structures might be influenced by the action of chromatin remodelers. This idea prompted us to investigate gene domain structures in chromatin remodeler mutants known to affect chromatin accessibility, such as *brahma* (*brm*) and the *chromatin-remodeling protein 11* (*chr11) chr17* double mutant lacking function of CHR11 and CHR17 (64–66). Single-gene domains of BRM- and CHR11-target genes were substantially altered in *brm* and *chr11 chr17* mutants, respectively (Figure 3K, M; Supplementary Figure S22), although expression levels of those target genes were not significantly changed (Figure 3L, N). Furthermore, single-gene domain structures were diminished for both up-regulated and down-regulated target genes in *chr11 chr17* mutant relative to wild type (Supplementary Figure S23), further supporting that chromatin remodelers contribute to establishing single-gene domains through regulating chromatin accessibility, rather than transcriptional activity. Taken together, these results indicate that accessible TSSs and TESs participate in the formation of single-gene domains.

### Binding of transcription factors enables the formation of single-gene domains

**Figure 4.**
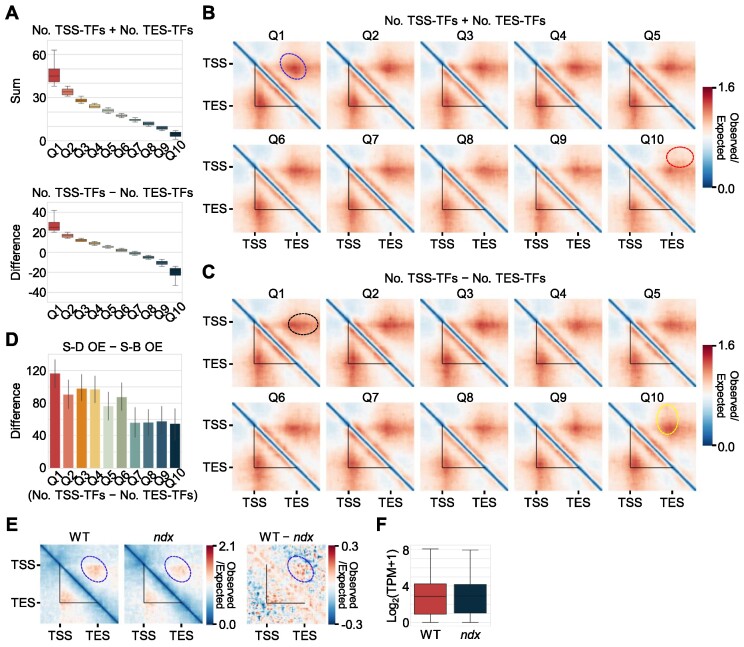
Transcription factors contribute to the formation of single-gene domains. (**A**) Sum (top) and difference (bottom) values of the numbers of TSS- and TES-binding transcription factors (TFs) measured by DAP-seq data. Genes longer than 2 kb were divided into 10 clusters according to the sum and difference of the numbers of transcription factors binding to the TSS and TES. The plots show the sum or difference of the numbers of transcription factors binding to the TSS and TES in each quantile. The box represents the interquartile range of the data, and the horizontal line indicates the median. The whiskers indicate 1.5 times the interquartile range. Outliers are not shown. (**B**) Pile-up images of Hi-C contact matrices of *Arabidopsis* genes in each quantile divided by the sum of the numbers of transcription factors binding to the TSS and TES of individual genes. (**C**) Pile-up images of Hi-C contact matrices of *Arabidopsis* genes in each quantile divided by the difference between the number of transcription factors binding to the TSS and the number of transcription factors binding to the TES of individual genes. Genes that have fewer than 10 transcription-factor-binding peaks were excluded from the analysis. Black circles indicate extended contacts between TSS and TES-downstream sequence, whereas yellow circles indicate extended contacts between TSS-upstream sequence and TES. (**D**) Difference of OE values at S-D and S-B subregions in each quantile divided by the difference between the numbers of transcription factors binding to TSSs and TESs of individual genes. The values are means, and the error bars indicate the 95% confidence interval. (**E**) Comparison of gene domain structures between wild type and *ndx* mutant. Domain structures of target genes of the transcription factor NDX (longer than 2 kb, *n* = 549) of *Arabidopsis* are shown. Left panel, wild-type Hi-C contact map; middle panel, Hi-C contact map of *ndx* mutant; right panel, difference in Hi-C contact maps between wild type and *ndx* mutant. (**F**) Expression levels of NDX-target genes (longer than 2 kb, *n* = 549) in wild type and *ndx* mutant. Log_2_(transcripts per million [TPM] + 1) values were used to represent expression values. In (B) and (E), the red circles indicate S-C subregion, whereas the blue circles indicate a TSS–TES contact site. In (B), (C) and (E), the black triangles indicate gene boundaries.

Transcription factors preferentially bind to accessible chromatin (67–69). We therefore tested whether the binding of transcription factors could promote the formation of single-gene domains. We employed DNA affinity purification and sequencing (DAP-seq) data for 470 *Arabidopsis* transcription factors to estimate transcription-factor-binding frequencies for all individual genes (47). We identified genes with associated DAP-seq peaks around their TSS or TES regions (100 bp on either side of the TSS or TES) and counted the number of transcription factors binding to each gene TSS and/or TES. Pile-up analysis revealed that the total number of transcription factors binding to the TSS and TES of each gene was positively correlated with the strength of single-gene domains (Figure 4A, B). Furthermore, genes with preferential binding of transcription factors at their TSSs showed strong contacts between their TSSs and the sequences downstream of their TESs (a black circle; S-D regions in the Hi-C contact map), whereas genes with more transcription factors binding to their TESs showed strong contacts between their TESs and the sequences upstream of their TSSs (a yellow circle; S-B regions in Hi-C contact map) (Figure 4C, D). Consistently, transcription factor binding frequency correlated to chromatin accessibility at TSS and TES, but showed a marginal correlation to gene expression levels in *Arabidopsis* (Supplementary Figure S24). We observed the similar association between transcription factor binding frequency and single-gene domain, independent on gene expression levels, in maize (Supplementary Figure S25). Genes having higher transcription factor binding frequency with a similar degree of chromatin accessibility exhibited a stronger gene domain structure (Supplementary Figure S26). These results suggest that transcription factor binding at accessible TSS and/or TES regions drives gene domain formation.

To validate these observations, we analyzed single-gene domain structures in mutants lacking a single transcription factor, such as the *ndx* (*nodulin homeobox*) mutant of *Arabidopsis* and the *tcp1* mutant of *M. polymorpha* (23,43). Single-gene domain structures of NDX-target genes were altered in the *ndx* mutant even with negligible changes in their gene expression levels, whereas nontarget genes did not show substantial alterations in single-gene domain structures (Figure 4E, F; Supplementary Figure S27A, B). Unexpectedly, in the *M. polymorpha tcp1* mutant, chromatin contacts in genic regions relative to surrounding regions were decreased in both TCP-target and nontarget genes, possibly owing to the widespread role of TCP1 in 3D chromatin architecture of the *M. polymorpha* genome (23) (Supplementary Figure S27C-E). Considering that TCP1-rich TADs were not substantially affected in the *tcp1* mutant (23), TCP1 might have a more profound influence on the establishment of gene domain structures in *M. polymorpha*. These results indicate that binding of transcription factors to TSSs and/or TESs is responsible for the formation of single-gene domains.

### Accessible TSSs and TESs of neighboring genes allow the formation of multigene domains

**Figure 5.**
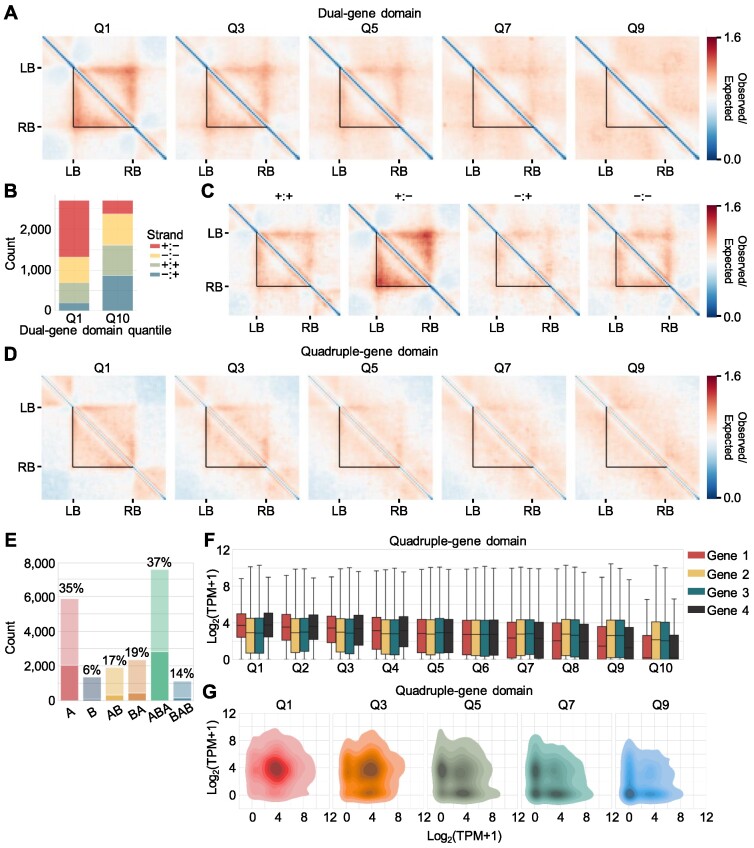
Multiple neighboring genes form multigene domains in *Arabidopsis*. (**A**) Pile-up images of Hi-C contact matrices of *Arabidopsis* dual-gene domains. Gene pairs were divided into 10 quantiles according to the values of left-border (LB) accessibility (TES or TSS) × right-border (RB) accessibility (TES or TSS). The pile-up images of Hi-C contact matrices of quantiles Q1, Q3, Q5, Q7 and Q9 are shown. (**B**) Distribution of gene pair orientation in quantiles Q1 and Q10 of dual-gene domains. The number of gene pairs in each orientation is shown. (**C**) Effect of gene pair orientation on dual-gene domain structures. Pile-up images of Hi-C contact matrices of *Arabidopsis* dual-gene domains with the top 20% boundary accessibility in each category are shown. The gene pair orientation is indicated above each image. (**D**) Pile-up images of Hi-C contact matrices of *Arabidopsis* quadruple-gene domains. All four nearby genes were divided into 10 quantiles according to the values of LB accessibility (TES or TSS) × RB accessibility (TES or TSS). Pile-up images of Hi-C contact matrices of quantiles Q1, Q3, Q5, Q7 and Q9 are shown. (**E**) Number of gene pairs with strong dual gene domains in each local A/B compartment defined at 5-restriction-fragment resolution. Gene pairs within Q1–Q3 quantiles clustered by LB accessibility × RB accessibility were annotated as strong dual-gene domains. Boxes with light colors indicate the total number of *Arabidopsis* gene pairs contained in each local A/B compartment. The ratio of the number of gene pairs with strong dual-gene domains over total number of gene pairs in each local compartment is provided. (**F**) Expression levels of component genes of quadruple-gene domains. The *x*-axis indicates the quantiles of quadruple-gene domains, which were divided by the values of left-border accessibility × right-border accessibility. **(G)** Density plots of the expression levels of gene pairs located at boundaries of quadruple-gene domain. The *x*-axis indicates the expression levels of genes located at the left border of quadruple-gene domains, and the *y*-axis indicates the expression levels of genes located at the right border of quadruple-gene domains. Log_2_(transcripts per million [TPM] + 1) values were used to represent expression values. In (A), (C) and (D), the black triangles indicate left and right domain boundaries. In (F), the box represents the interquartile range of the data, and the horizontal line indicates the median value. The whiskers indicate 1.5 times the interquartile range. Outliers are not shown.

We predominantly observed gene domains at the single-gene scale. However, genes with imbalanced accessibility between their TSSs and TESs exhibited extended interactions with surrounding regions outside TSS or TES (Figure 3G). Accordingly, multiple neighboring genes might form larger domains. We examined the formation of dual-gene domains consisting of two neighboring genes. We calculated the product of the TSS or TES accessibility of one gene (left border) and the TSS or TES accessibility of another gene (right border) for all gene pairs (*n* = 27 040) and clustered the gene pairs into 10 quantiles according to these product values (left-border accessibility × right-border accessibility) (Supplementary Figure S28A). Pairs of genes with high accessibility around their border regions exhibited a strong dual-gene domain structure (Figure 5A; Supplementary Figure S28B). While a gene pair located within a short linear gene-to-gene distance usually exhibited stronger domain structures, chromatin accessibility was still critical for dual-gene domain formation as shown by the same analysis performed with a linear distance cutoff (200 bp < distance between genes < 20 000 bp) (Supplementary Figure S29).

Furthermore, since TSSs preferentially interacted with their downstream regions and usually had higher accessibility than did TESs (Figure 3G; Supplementary Figure S15), convergent gene pairs exhibited stronger formation of dual-gene domains via highly accessible TSS-TSS anchors, whereas divergent gene pairs had weaker dual-gene domain structures (Figure 5B, C). Parallel gene pairs showed a moderate strength of dual-gene domain structures (Figure 5C). We also detected dual-gene domains in the tomato, maize and *M. polymorpha* genome, which were dependent on accessible TSSs or TESs around gene domain boundaries (Supplementary Figure S30). Accordingly, multigene domains may be established on the basis of accessibility of gene border regions in plant genomes.

We aimed to explore whether larger multigene domains might form with more than two neighboring genes. Hence, we grouped all possible combinations of three or four adjacent *Arabidopsis* genes into 10 quantiles according to the boundary accessibility at both ends (left-border accessibility × right-border accessibility). As with the formation of dual-gene domains, high border accessibility at the TSSs or TESs of boundary genes allowed the formation of multigene domains (Figure 5D; Supplementary Figure S31). Low accessibility of genes located in the middle of multigene domains also contributed to the formation of multigene domains (Supplementary Figure S32). Additionally, compartment analysis also revealed that strong multigene domains were formed either in local A or ABA compartment (Figure 5E), similar to single-gene domain structures (Figure 3H). These results indicate that accessible regions at the TSSs or TESs of boundary genes are anchoring sites, and contacts between accessible gene borders enable the formation of multigene domains in *Arabidopsis*.

The formation of multigene domains raised the possibility that their structure might also be associated with transcriptional regulation of their constituent genes. Pairs of genes located at accessible boundaries of a multigene domain were expressed at a high level due to their high chromatin accessibility compared with the lowly accessible genes located at the middle regions of a multigene domain (Figure 5F; Supplementary Figure S33). This observation is consistent with previous results that genes located at TAD boundaries, but not those located inside of TADs, were highly expressed (1,70,71). In addition to expression levels, gene pairs at the boundary of a multigene domain that were in contact tended to be highly expressed together (Figure 5G; Supplementary Figure S34). Despite the co-expression of the boundary genes in certain conditions, they were not generally co-regulated in response to certain stimuli (Supplementary Figure S35), which suggests that multigene domain structures dynamically change in response to internal and external stimuli, possibly to reestablish co-expressed gene pairs.

### Accessible TSSs and TESs participate in the formation of basic building blocks of chromatin architecture in *Arabidopsis*

**Figure 6.**
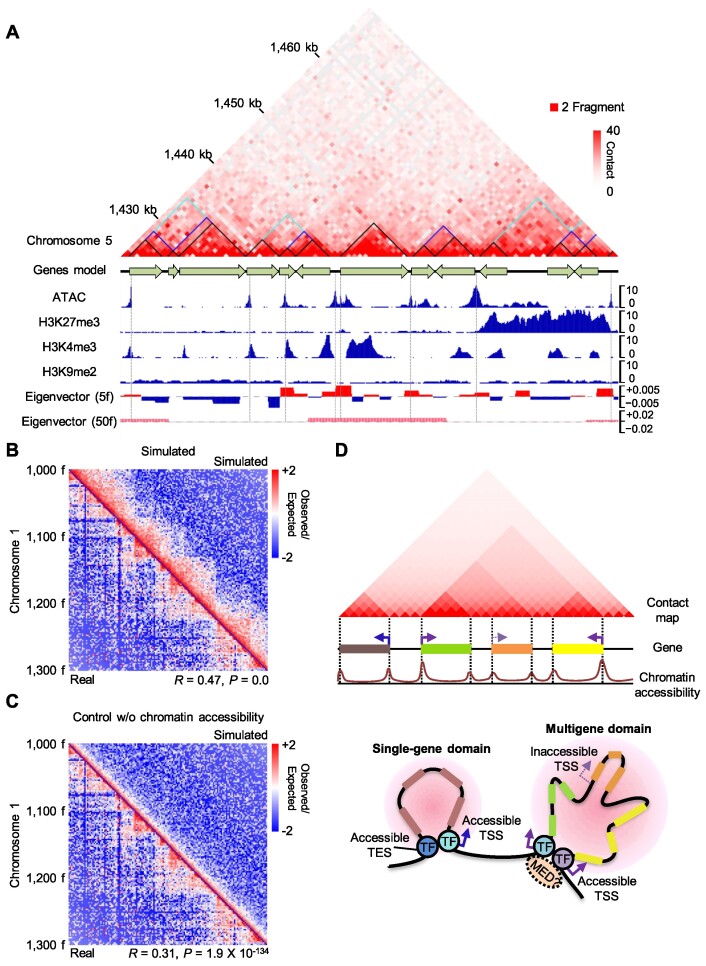
Accessible gene boundaries are basic units for local chromatin architecture. (**A**) A KR-normalized Hi-C contact map with chromatin status. Black triangles in the Hi-C contact map indicate individual genes. Blue and sky blue triangles in the Hi-C contact map indicate dual-gene domains (quantiles Q1–Q4; values of left-border accessibility × right-border accessibility / multiplied values of internal accessibility) and triple-gene domains (quantiles Q1–Q3; values of left-border accessibility × right-border accessibility / multiplied values of internal accessibility), respectively. The first eigenvector (A/B compartment) was calculated at 5-restriction-fragment and 50-restriction-fragment resolution. (B, C) Simulated Hi-C contact maps at two-restriction-fragment resolution with (**B**) or without (**C**) chromatin accessibility information. The upper-right triangle image shows the simulated OE contact map, while the lower-left triangle image shows the real OE contact map. Spearman's correlation coefficient with *P*-value is indicated. Interacting bins (<50 fragments in gap distance between anchors) were used for the correlation coefficient calculation. (**D**) Diagram of the proposed model. Transcription factors (TFs) that bind to accessible TSSs and/or TESs establish gene domains, basic structural units of chromatin architecture in *Arabidopsis*. Mediator (MED) proteins may act as physical interaction links between TFs. The gene domains contribute to establishing global 3D folding structures in plant genomes.

Our results demonstrate that single genes as well as multiple neighboring genes with accessible domain boundaries (TSSs or TESs) tend to form a regular chromatin domain structure. Since genes are densely distributed across the *Arabidopsis* genome, we hypothesized that accessible TSSs and TESs contribute to forming a basic unit for 3D chromatin architecture of the *Arabidopsis* genome. Most boundaries of 3D interacting units in raw Hi-C contact maps matched the boundaries of gene domains, which also significantly overlapped with accessible chromatin regions and local A compartments (Figure 6A; Supplementary Figure S36).

To validate our hypothesis, we built a model using chromatin accessibility at gene boundary regions, gene position, and gene orientation information to simulate high-resolution Hi-C contact maps. We added accessibility-dependent contact values to a distance-decay backbone model and considered directionality of chromatin contacts (TSSs with downstream contacts and TESs with upstream contacts; Materials and Methods). As a result, we successfully simulated local Hi-C contact maps at a resolution of two restriction fragments with reasonable Spearman's correlation coefficients, which were higher than those simulated only with the backbone distance-decay model (Figure 6B, C; Supplementary Figure S37A–D). Broader regions of the Hi-C maps were also reasonably simulated with our chromatin-accessibility-dependent model (Supplementary Figure S37E). These results indicate that accessibility at TSSs and TESs is a key determinant of local 3D chromatin architecture, at least for the *Arabidopsis* genome.

## DISCUSSION

TAD-like structures identified in the genomes of plant species (15–18,20,22,23) are different from canonical TADs of mammalian genomes. The most profound difference is the lack of strong contacts at domain boundaries in plant Hi-C contact maps (15,16), possibly owing to the absence of architectural proteins. Instead, plant-specific TADs are more dependent on local chromatin states (15,16), similar to compartmental domains of animals (13). Even in the *Arabidopsis* genome that lacks obvious TAD or TAD-like structures, compartment-dependent chromatin interactions are likely relevant in establishing 3D chromatin architecture, as exemplified by Polycomb-associated domains (33). Therefore, chromatin-state-dependent formation of a 3D genome architecture is likely prevalently conserved in plants, although the precise nature of the basic structural unit and its formation mechanism have remained unknown.

When reanalyzing deeply sequenced Hi-C data available for the *Arabidopsis* genome at single-restriction-fragment resolution, we discovered gene domain structures consisting of single or multiple neighboring genes. The accessible TSS and/or TES regions of these genes serve as anchoring sites for single-gene and multigene domains. The formation of gene domains contributes to the control of gene expression and the establishment of a core building block for 3D chromatin architecture. The single-gene domain described in this study shares structural features with the gene mini-domains identified in the *D. melanogaster* genome in that both exhibit contact enrichment within gene bodies in Hi-C contact maps (12). These domain structures are also associated with the transcriptional activity of genes, as shown by the strong formation of domains around highly expressed genes (12). Despite their structural similarities, the molecular mechanisms for domain formation are different in plants and *D. melanogaster*: gene mini-domains are independent of chromatin accessibility (12), whereas single-gene domains are established by accessible TSS or TES regions. Gene domains should also be distinguished from gene loops. Whereas gene loops commonly include chromatin loops formed within an individual gene, including promoter-terminator (31,72,73), TSS–TES (29,30), and intragenic loops (32,74), single-gene domains are mainly established by TSS–TES contacts. Although it may be hard to distinguish between single-gene domains and gene loops linking TSSs and TESs, the single-gene domain should be conceptually distinguished as a 3D chromatin contact unit that exhibits contact enrichment within whole gene body region in raw Hi-C contact maps (Supplementary Figure S36). It is also noteworthy that the gene domains can be expanded to two genes or more by anchoring the TSS or TES of the boundary gene at each end of the domain. Therefore, a gene domain can be defined as a local chromatin contact domain anchored by accessible TSS/TES regions.

We showed that gene domains, which are formed at single- and multigene scales, are widespread and exist across the entire genome. In addition, they are smaller than TADs or compartmental domains and thus likely to act as basic structural units of genome-wide chromatin architecture. Consistently, in tomato, maize and *M. polymorpha* genomes, strongly accessible gene borders significantly overlapped with conventional TAD boundaries identified at 5 kb resolution (Supplementary Figure S38). Furthermore, strong single-gene domains were formed within both A and B compartments defined at 50-restriction fragment resolution (Supplementary Figure S20), suggesting that the formation of single-gene domain occurs likely independent of conventional large A/B compartments. At higher 5-restriction fragment resolution, the local A/B compartments smaller than a gene scale showed distinct chromatin features from conventional large A/B compartments. Surprisingly, the local A/B compartments were mainly determined by chromatin accessibility (Figure 3I, J; Supplementary Figure S21), unlike conventional large A/B compartments established primarily by epigenetic states. Consistently, accessible gene borders were mainly annotated as local A compartments in contrast to gene bodies. In agreement with gene domain conformation, strong gene domains predominantly formed in the local ABA compartment (Figure 3H). Given that B compartment domains are frequently enclosed by A compartment domains and that many gene-to-gene loops spanning intergenic regions exist in the large genomes of plants (15,17,22), gene domain formation mediated by accessible gene borders would be a general mechanism to establish a basic 3D folding unit in plants.

Various types of 3D chromatin structures are indeed associated with accessible gene boundaries, as we largely recapitulated Hi-C contact maps by computational simulation using chromatin accessibility of TSSs and TESs. The presence of gene domains is not limited to the *Arabidopsis* genome, as we also detected them in the genomes of *M. polymorpha*, tomato, and maize, indicating that the contribution of gene domains to establishing 3D chromatin architectures might be conserved across the plant lineage.

Local chromatin architectures are dependent on accessible gene boundaries, which are mainly targeted by transcription factors. Thus, we propose that transcription factors may participate in the formation of gene domains and thus local 3D chromatin folding (Figure 6D). This notion is supported by the observation that the number of transcription factors that bind to the TSS and/or TES of each gene, which may reflect DNA-anchoring frequency, is correlated with the strength of gene domain formation (Figure 4A, B). Thus, the huge collection of transcription factors typically coexisting within plant cells may shape genome-wide chromatin architectures, demonstrating that gene domains are mainly established by protein–protein interactions among transcription factors binding to TSSs/TESs, rather than by orthologs of architectural proteins like CTCF and cohesin. In agreement with this idea, short-range looping of accessible chromatin regions via binding of transcription factors has also been identified in humans (75). The transcription factors ZNF143 (ZINC FINGER PROTEIN 143) and HCFC1 (HOST CELL FACTOR C1) mediate such short-range chromatin loops independently of CTCF, supporting our model wherein transcription factors are responsible for local 3D chromatin organization (75).

Although we extensively characterized gene domain structures, several structural features remain to be fully elucidated. In general, TSSs and TESs have opposite directionality in chromatin contacts: TSSs preferentially interact with downstream regions, whereas TESs interact with upstream regions. Our simulation model should consider directional interactions of TSSs and TESs in predicting 3D chromatin configurations. However, what is responsible for this observed biased contact directionality of TSSs and TESs is unclear. A possible mechanism is the opposite orientation of transcription-factor-binding motifs at each end of accessible gene boundaries, similar to the formation of canonical TADs in mammals that is dependent on CTCF motifs in a convergent orientation (76). In addition, gene domains have relatively weak anchoring strength at boundary regions, as only distance-normalized Hi-C contact maps, but not raw Hi-C maps, exhibit anchoring spots in plant genomes, in contrast to the CTCF anchors of canonical TADs that can be seen even in raw Hi-C contact maps. Our simulation model also included an accessibility-dependent term that follows a slower distance-decay rate than that of the backbone model. These results suggest that anchors of gene domains in plant genomes are not as tightly tethered as CTCF loops but rather weakly bound together through interactions among proteins, including transcription factors, mediators, transcription complex components, and RNA polymerase II, that bind to accessible TSS/TES regions. Given that accessible gene borders are responsible for the local chromatin architecture of the genomes of various plant species, techniques such as highly sensitive transposase-mediated analysis of chromatin, which enables the detection of chromatin loops between accessible chromatin regions at higher resolution (75), may be applied to further understand the principles and molecular mechanism underlying 3D chromatin folding in plants.

## Supplementary Material

gkad710_Supplemental_Files

## Data Availability

All public datasets used in this study are listed in Supplementary Table S1. Raw data and all implemented codes used in this study have been deposited in figshare repository (DOI: 10.6084/m9.figshare.23290613).
