# Supplementary material for: Accessible gene borders establish a core structural unit for chromatin architecture in Arabidopsis
Source: Nucleic Acids Res. 2023 Sep 1;51(19):10261–77. doi: 10.1093/nar/gkad710 (PMC10602878; doi:10.1093/nar/gkad710)
Supplement: gkad710_Supplemental_Files [file gkad710_supplemental_files.zip › Supplementary Figures.pdf]

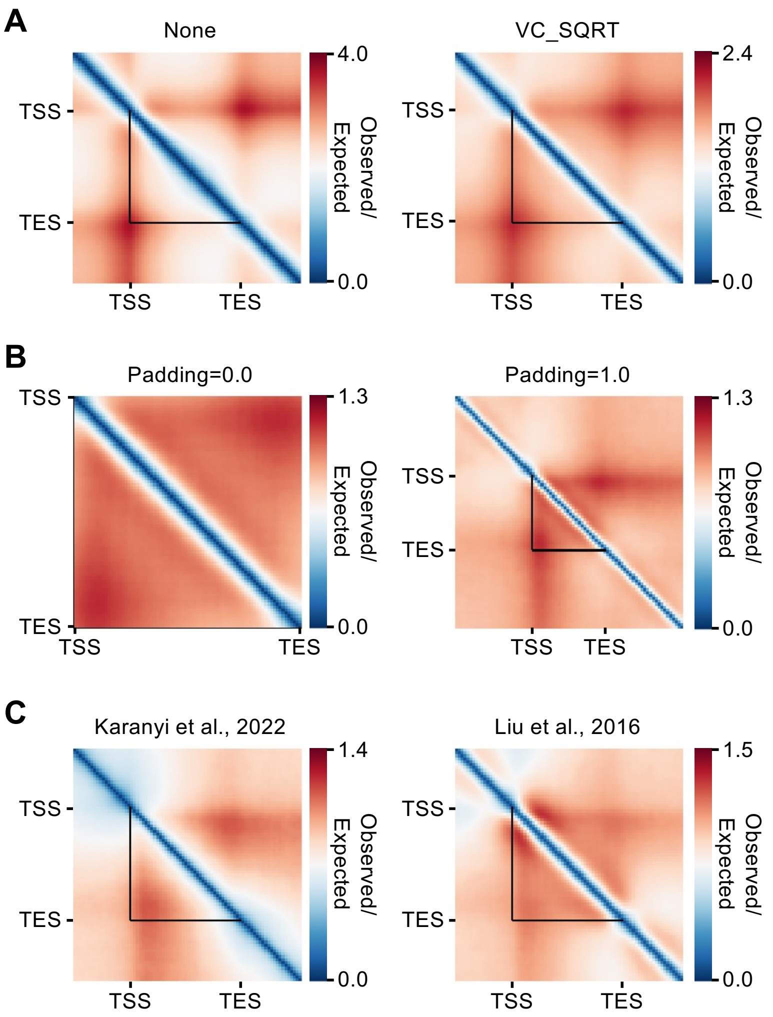

Supplementary Figure S1. Single-gene domain structures in *Arabidopsis.*

(**A–C**) Pile-up images of Hi-C contact matrices with different normalization methods (**A**), padding sizes (**B**), and datasets (**C**). Whole genes were used for the pile-up analysis. Black triangles indicate gene boundaries. (**A**) Pile-up images of unnormalized contact matrix (left) or VC_SQRT-normalized contact matrix (right). (**B**) Pile-up images of Hi-C contact matrices with padding sizes of gene length multiplied by 0.0 (left) or 1.0 (right). (**C**) Pile-up images of Hi-C contact matrices with the data from Karanyi et al., 2022 (left), or Liu et al., 2016 (right).

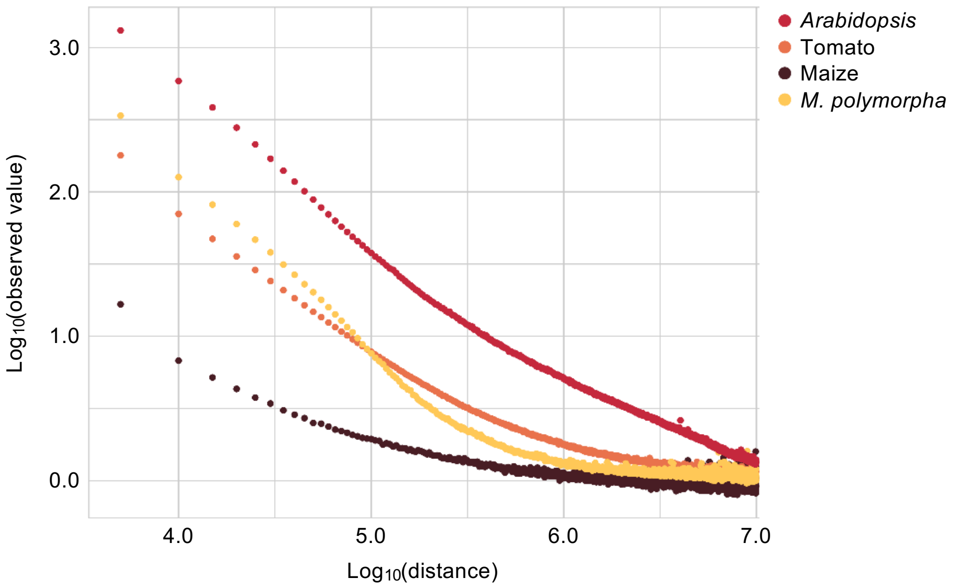

Supplementary Figure S2. Distance-decay models of Hi-C data from various plant species. Scatterplot representing the relationship between $\log_{10}$(distance) and $\log_{10}$(observed value of a paired end Hi-C read). The *x*-axis indicates log-transformed distances between two bins. The *y*-axis indicates log-transformed KR-normalized observed values. KR-normalized observed values at 5 kb resolution for chromosome 1 from each plant species were used in the analysis. Each point indicates the average KR-normalized observed value at a given distance.

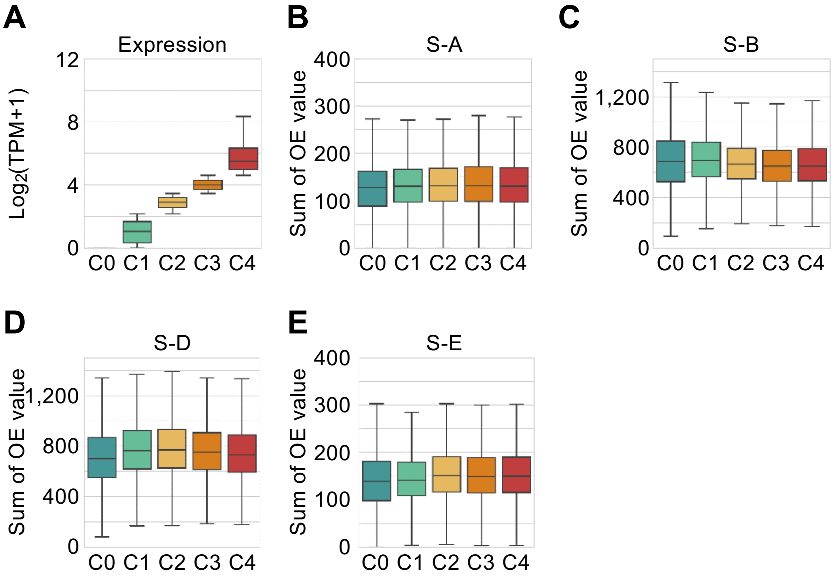

Supplementary Figure S3. Relationship between single-gene domains and gene expression in *Arabidopsis.*

(**A**) Gene expression levels in each expression quantile. Genes longer than 2 kb (n = 14,411) were divided into five clusters according to their expression levels from C0 (not expressed) to C4 (highly expressed). (**B**–**E**) Sum of chromatin contacts (observed/expected [OE] of a paired end Hi-C read) within the subregions Surrounding-A (S-A) (**B**), S-B (**C**), S-D (**D**), and S-E (**E**) in each expression quantile of *Arabidopsis* genes. The box represents the interquartile range of the data, and the horizontal line indicates the median value. The whiskers indicate 1.5 times the interquartile range. Outliers are not shown.

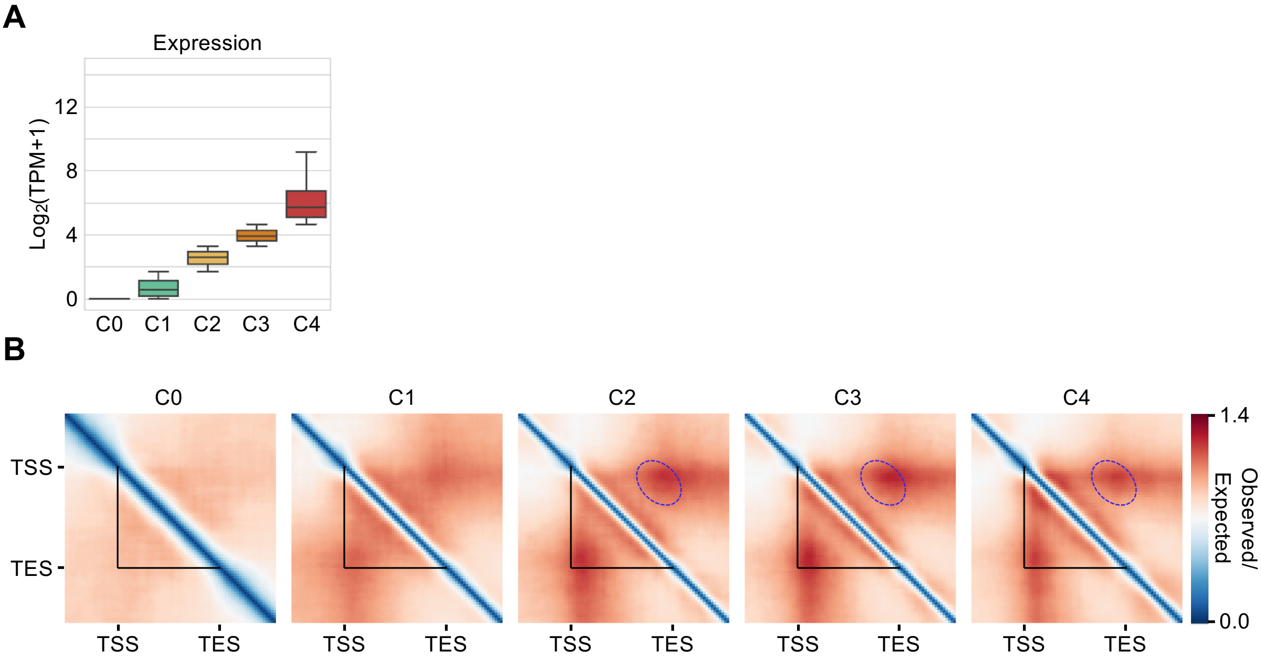

Supplementary Figure S4. Correlation between single-gene domains and gene expression in *Arabidopsis.*

(**A**) Gene expression levels in each expression quantile. The box represents the interquartile range of the data, and the horizontal line indicates the median value. The whiskers indicate 1.5 times the interquartile range. Outliers are not shown. (**B**) Pile-up images of Hi-C contact matrices for *Arabidopsis* genes clustered according to their expression levels from C0 (not expressed) to C4 (highly expressed). Black triangles indicate gene boundaries. In (**A**) and (**B**), all genes without length cutoff were used for the analysis.

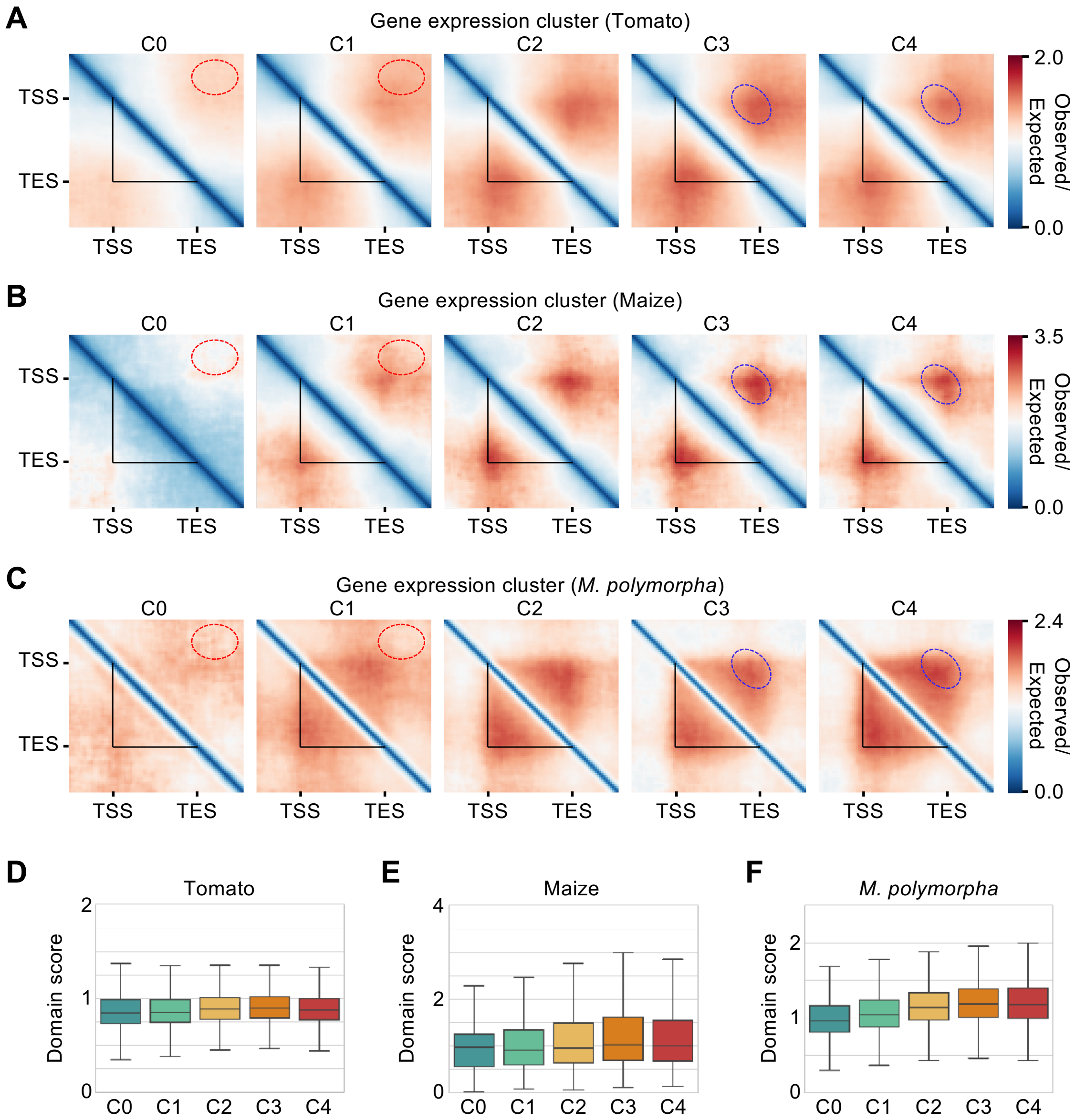

Supplementary Figure S5. Correlation between single-gene domains and gene expression in several plant species.

(**A**−**C**) Pile-up images of Hi-C contact matrices of genes from tomato (**A**), maize (**B**) and *M. polymorpha* (**C**). Genes longer than 2 kb were divided into five clusters according to their expression levels from C0 (not expressed) to C4 (highly expressed). Black triangles indicate gene boundaries. Red circles indicate S-C subregion, whereas blue circles indicate a TSS–TES contact site. (**D**−**F**) Domain score (DS) of genes in each expression quantile for tomato (**D**), maize (**E**) and *M. polymorpha* (**F**). DS was calculated as the mean G contact value (OE/pixel) divided by the mean S-C contact value (OE/pixel). The box represents the interquartile range of the data, and the horizontal line indicates the median value. The whiskers indicate 1.5 times the interquartile range. Outliers are not shown.

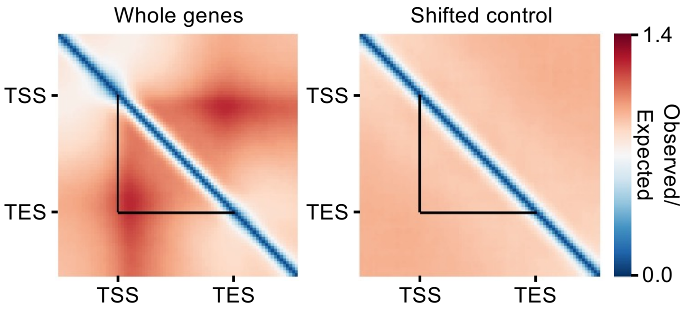

Supplementary Figure S6. Single-gene domain structures upon heat exposure in the *Arabidopsis* genome.

Pile-up images of Hi-C contact matrices of all *Arabidopsis* genes under heat condition (left). The same analysis was performed for all 5 kb upstream regions of all individual genes as a control (right, shifted control). Black triangles indicate gene boundaries.

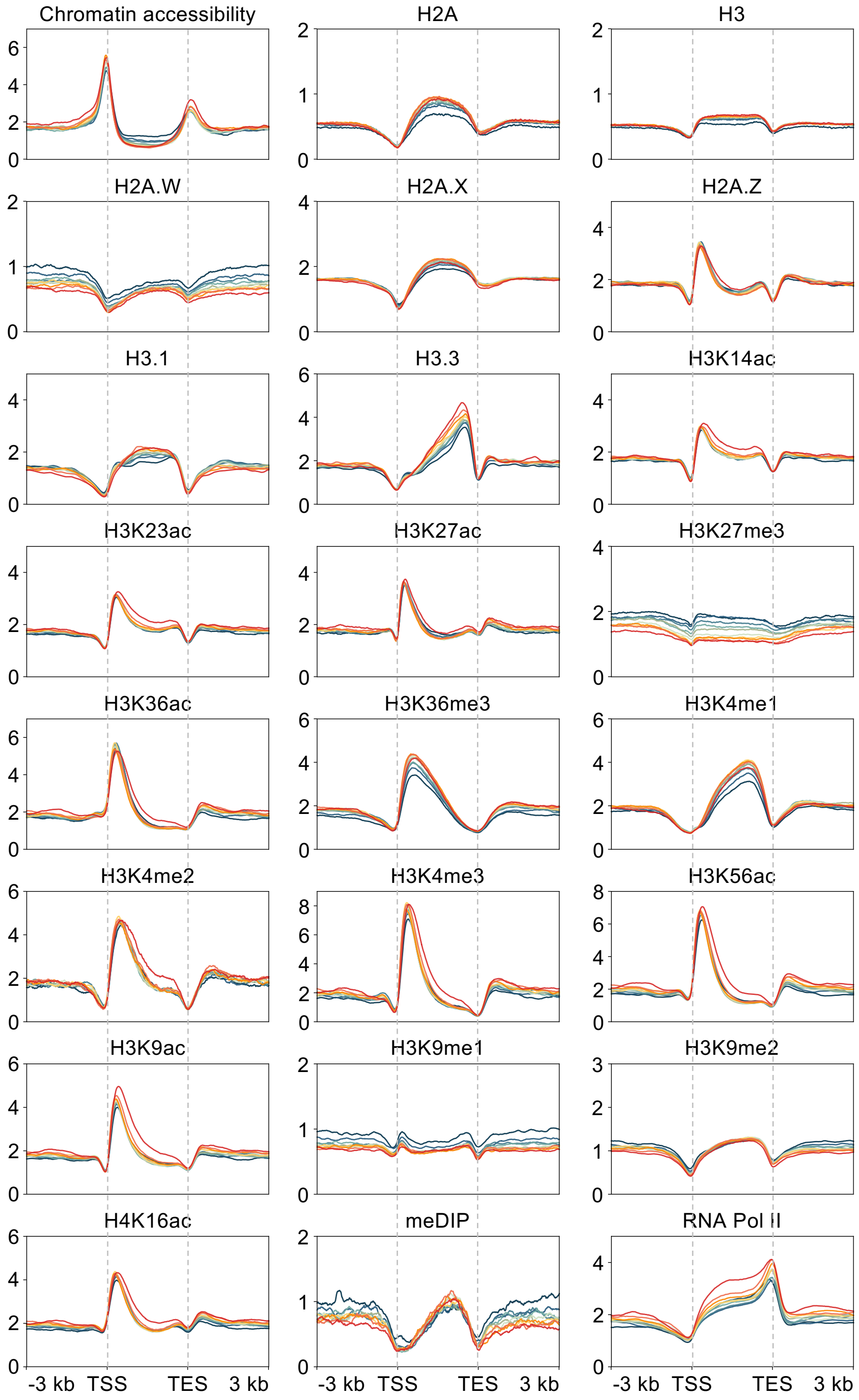

Supplementary Figure S7. Epigenetic profiles in quantiles divided by contact values in the gene body region.

Metaplots of 24 chromatin features in each quantile. Genes longer than 2 kb were divided into 10 quantiles according to the contact values (observed/expected values) within the gene body region.

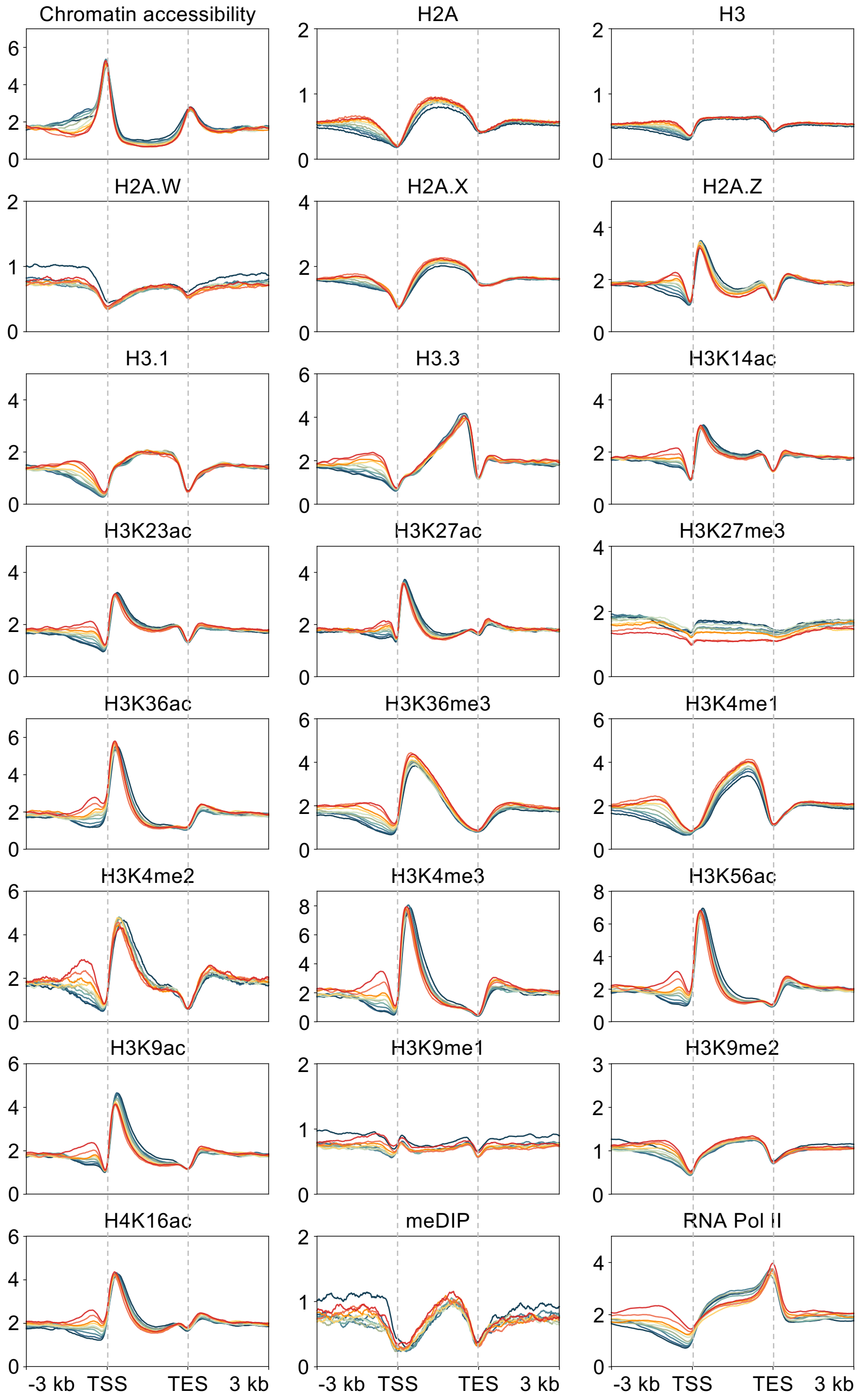

Supplementary Figure S8. Epigenetic profiles in quantiles divided by contact values in the S-A region.

Metaplots of 24 chromatin features in each quantile. Genes longer than 2 kb were divided into 10 quantiles according to the contact values (observed/expected values) within the S-A region.

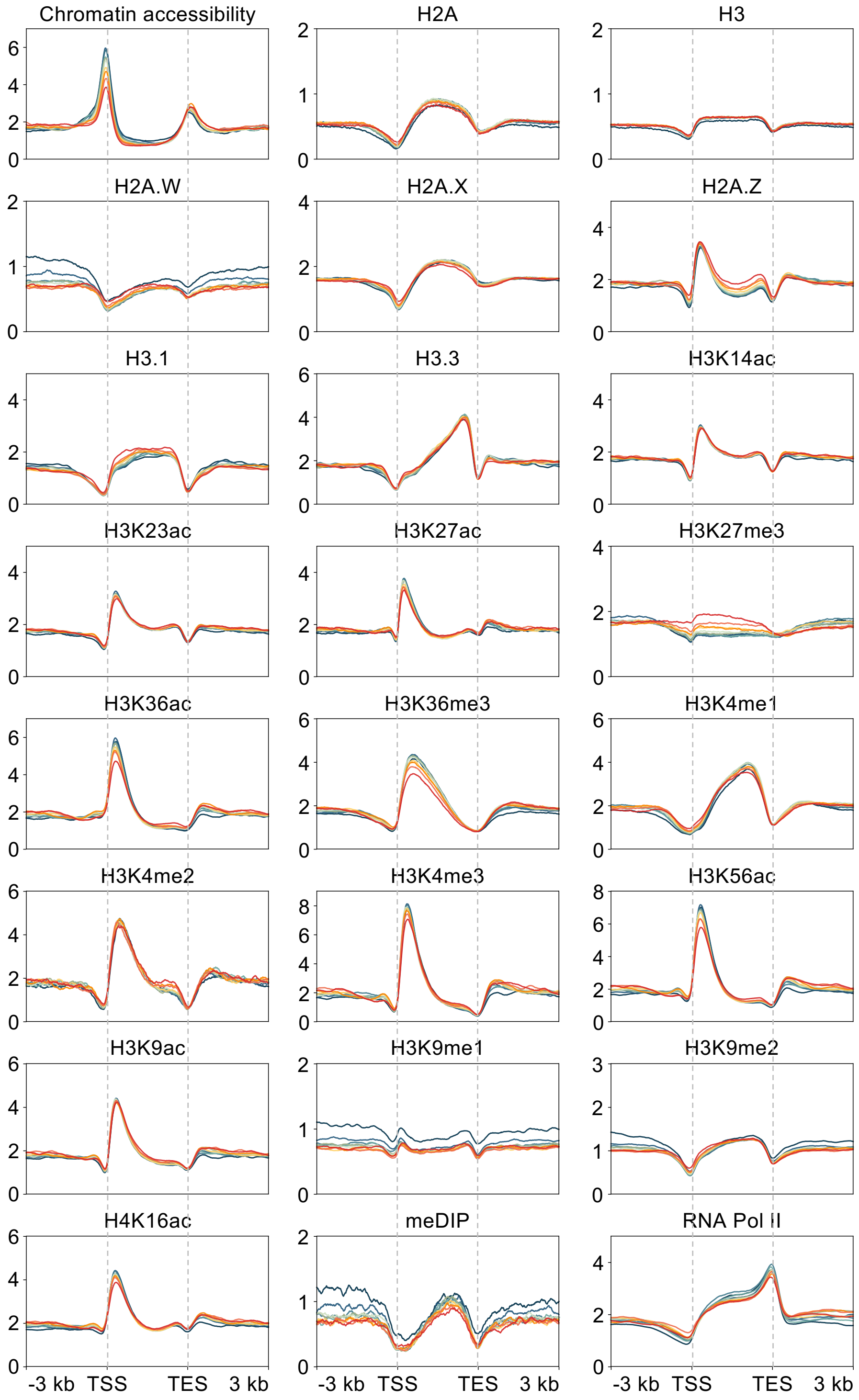

Supplementary Figure S9. Epigenetic profiles in quantiles divided by contact values in the S-B region.

Metaplots of 24 chromatin features in each quantile. Genes longer than 2 kb were divided into 10 quantiles according to the contact values (observed/expected values) within the S-B region.

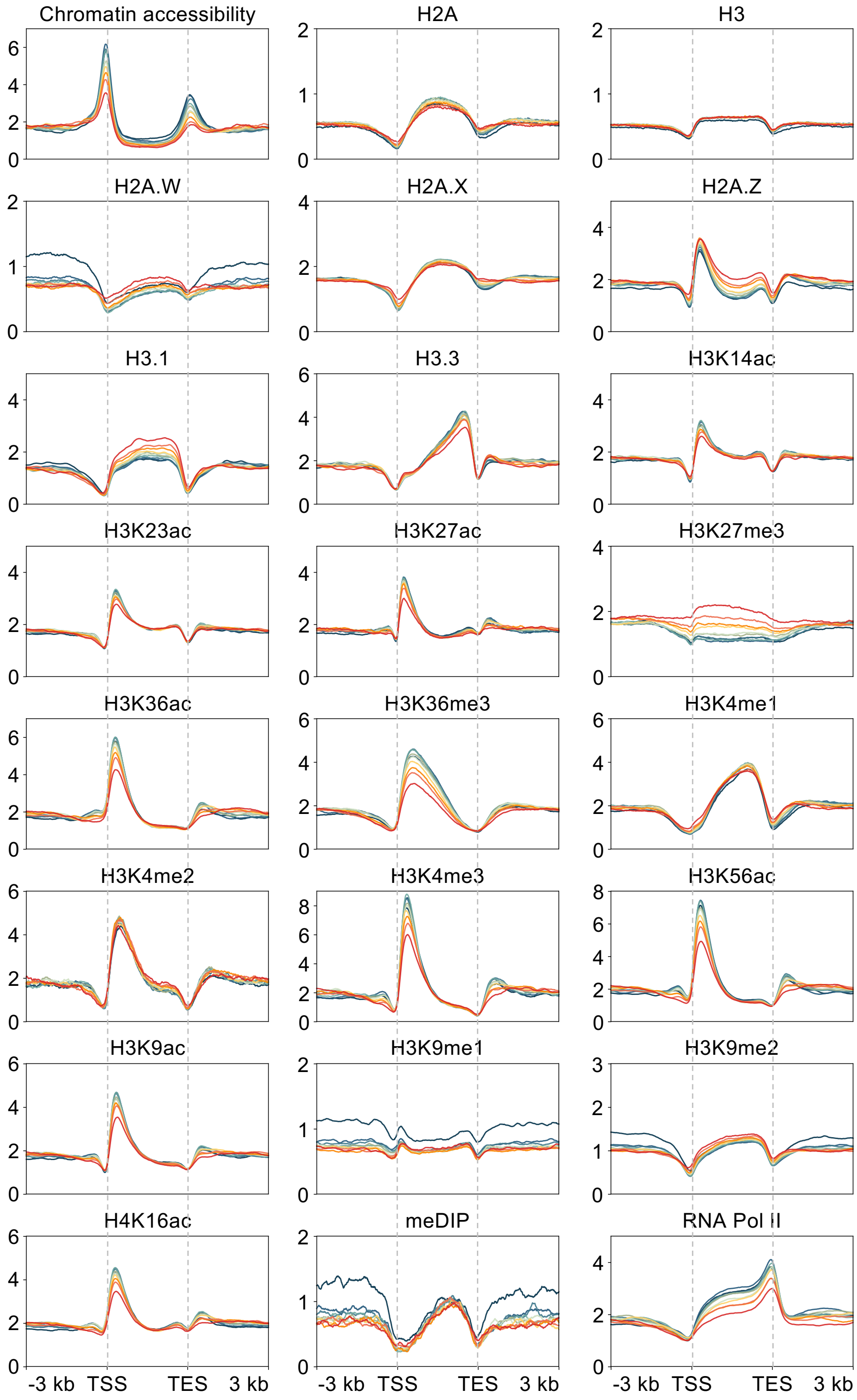

Supplementary Figure S10. Epigenetic profiles in quantiles divided by contact values in the S-C region.

Metaplots of 24 chromatin features in each quantile. Genes longer than 2 kb were divided into 10 quantiles according to the contact values (observed/expected values) within the S-C region.

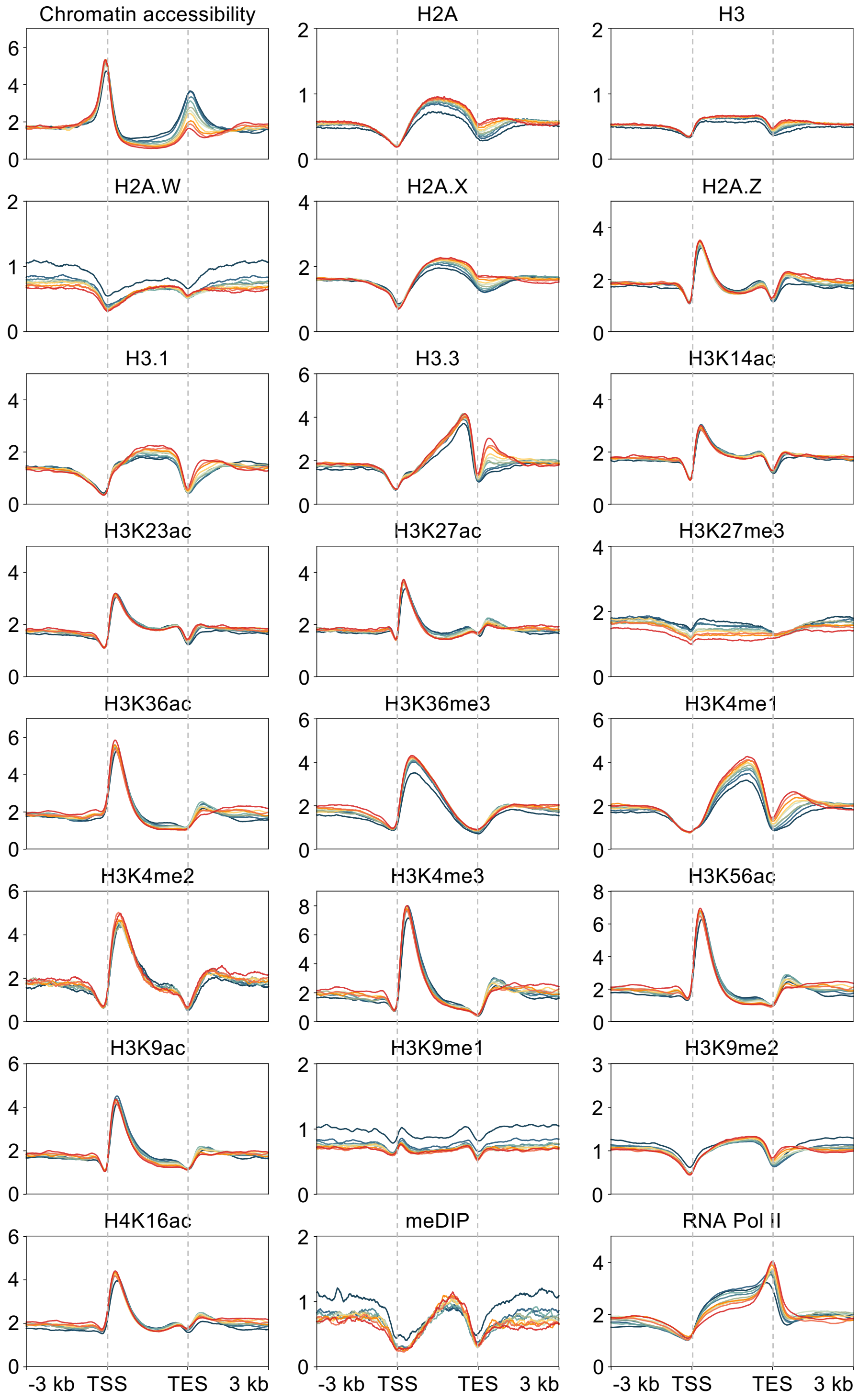

Supplementary Figure S11. Epigenetic profiles in quantiles divided by contact values in the S-D region.

Metaplots of 24 chromatin features in each quantile. Genes longer than 2 kb were divided into 10 quantiles according to the contact values (observed/expected values) within the S-D region.

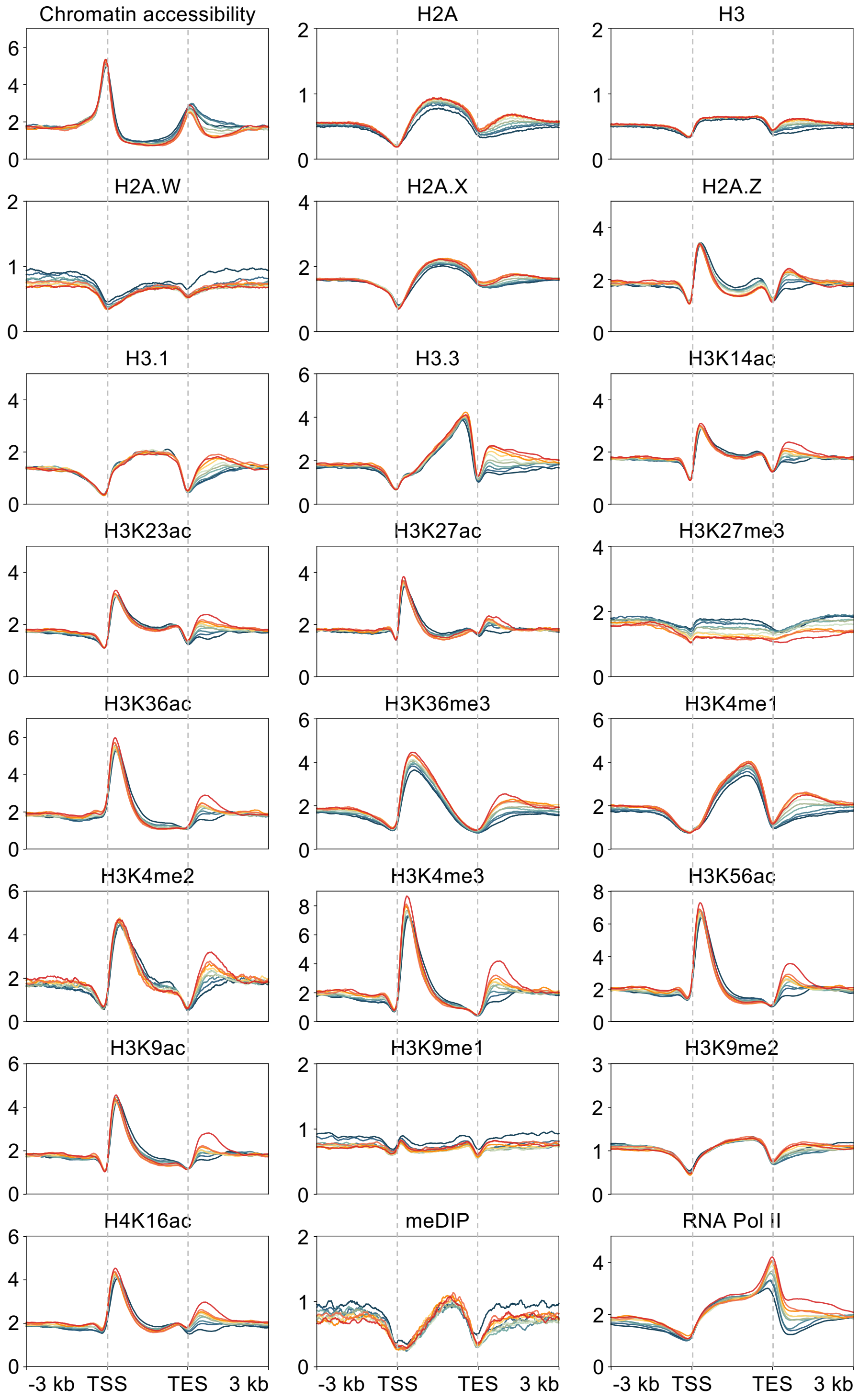

Supplementary Figure S12. Epigenetic profiles in quantiles divided by contact values in the S-E region.

Metaplots of 24 chromatin features in each quantile. Genes longer than 2 kb were divided into 10 quantiles according to the contact values (observed/expected values) within the S-E region.

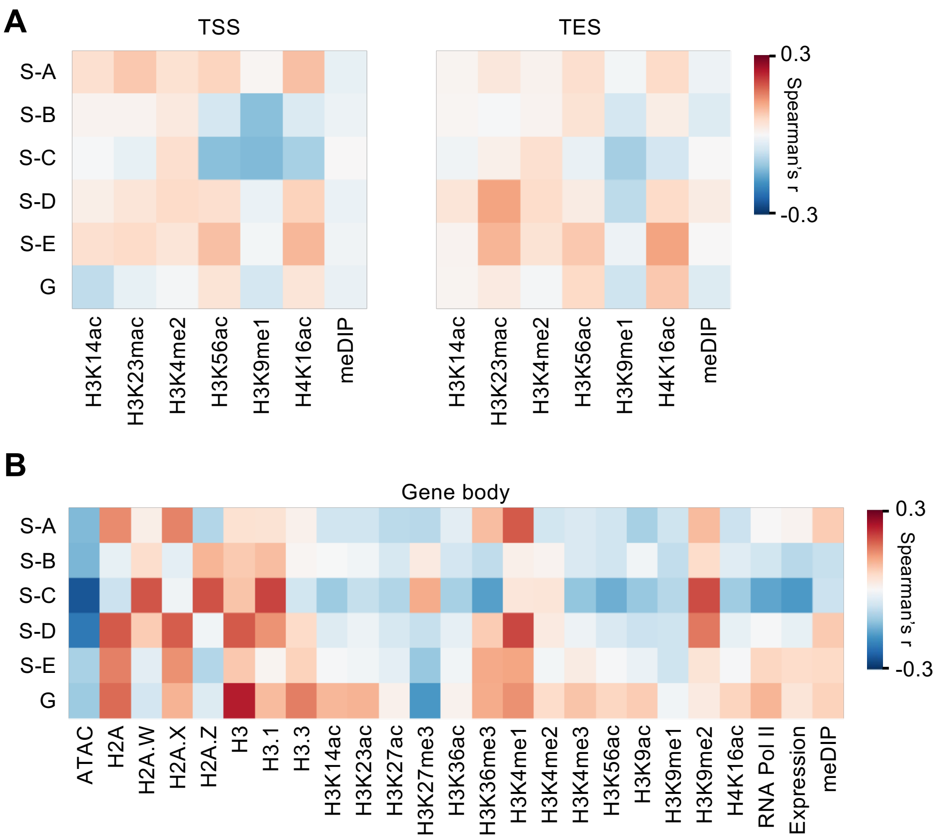

Supplementary Figure S13. Correlation analysis between subregional chromatin contacts and chromatin features.

(**A**, **B**) Correlation heatmaps between the sum of chromatin contacts at each subregion and the enrichment of chromatin features. Enrichment levels of chromatin features at TSS (**A**), TES (**A**), and gene body (**B**) regions were used for the correlation analysis. Spearman's correlation coefficients are shown.

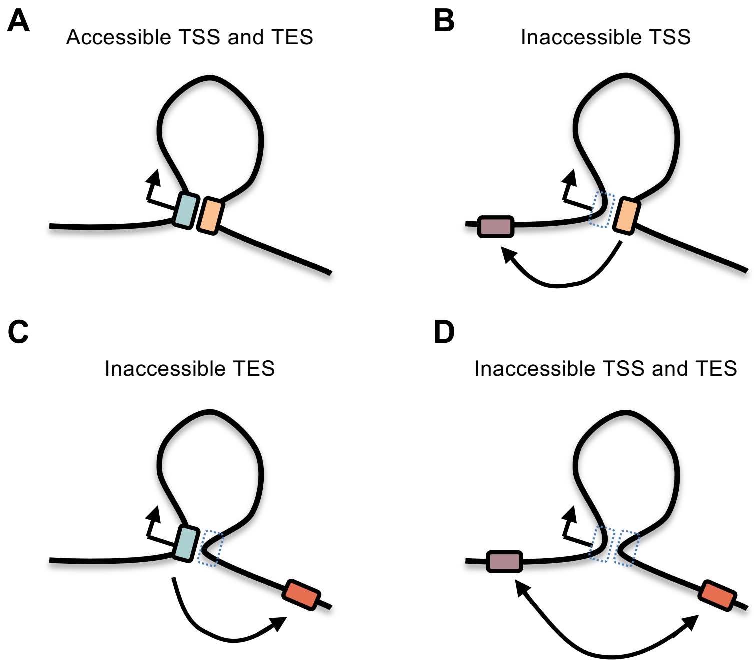

Supplementary Figure S14. Models of accessible gene border-dependent chromatin contacts.
(**A**) Chromatin contact model of genes with accessible TSS and TES. (**B**, **C**) Chromatin contact model of genes with inaccessible TSS (**B**) or inaccessible TES (**C**). (**D**) Chromatin contact model of genes with inaccessible TSS and TES. In (**A**−**D**), rectangles represent the accessible chromatin region, while dashed boxes represent the inaccessible gene border.

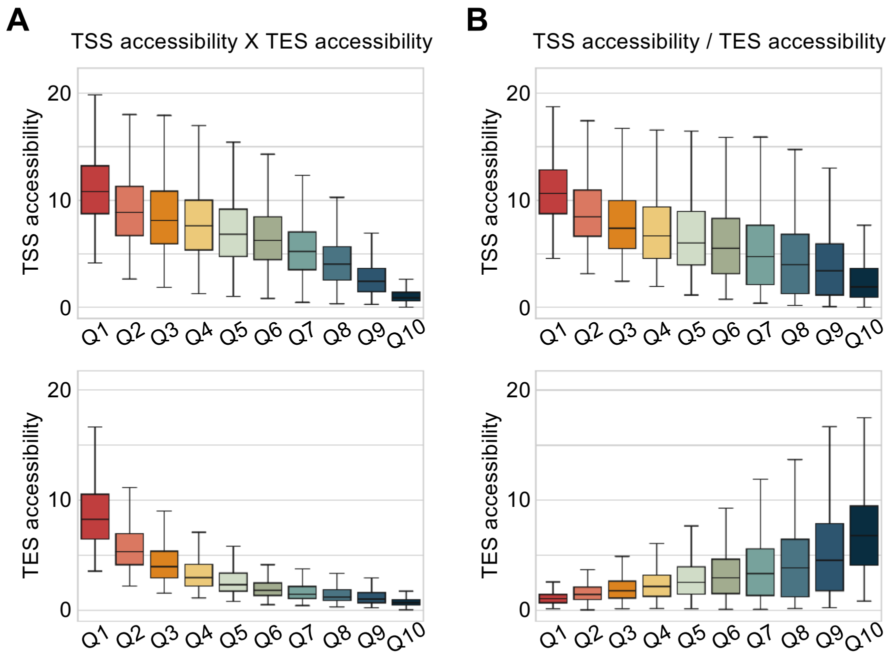

Supplementary Figure S15. Chromatin accessibility at the TSS and TES for each quantile of TSS accessibility × TES accessibility and TSS accessibility / TES accessibility.

(**A**, **B**) Chromatin accessibility in the quantiles divided by TSS accessibility × TES accessibility (**A**) or by TSS accessibility / TES accessibility (**B**). Genes longer than 2 kb were used for the analysis. The box represents the interquartile range of the data, and the horizontal line indicates the median value. The whiskers indicate 1.5 times the interquartile range. Outliers are not shown.

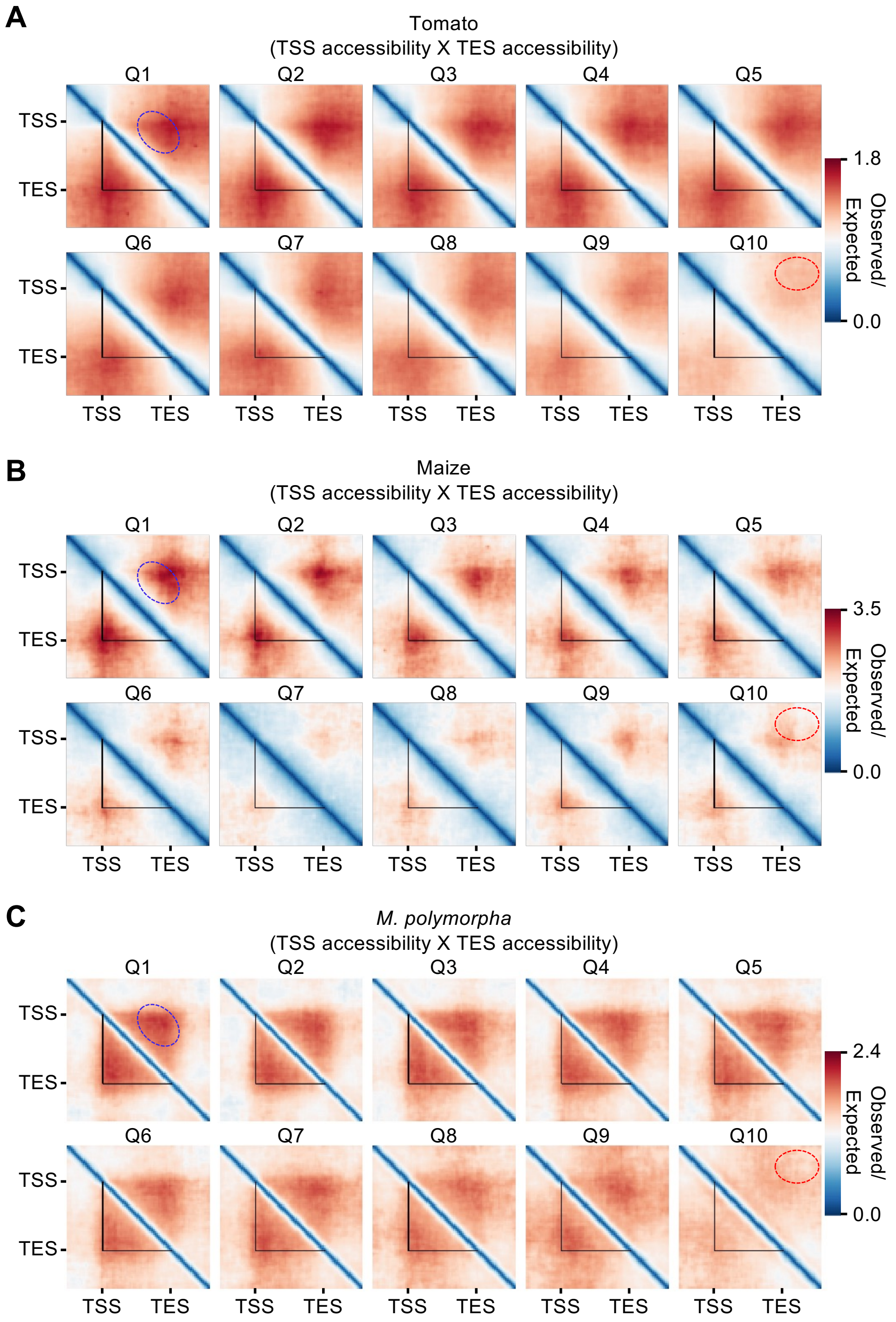

Supplementary Figure S16. Chromatin-accessibility-dependent formation of gene domains in several plant species.

(**A**−**C**) Single-gene domain structures in tomato (**A**), maize (**B**) and *M. polymorpha* (**C**). Genes longer than 2 kb were divided into 10 quantiles according to their TSS accessibility value × TES accessibility value. Pile-up images of Hi-C contact matrices in each quantile are shown. Black triangles indicate gene boundaries. Blue circles indicate a TSS–TES contact site, whereas red circles indicate S-C subregion.

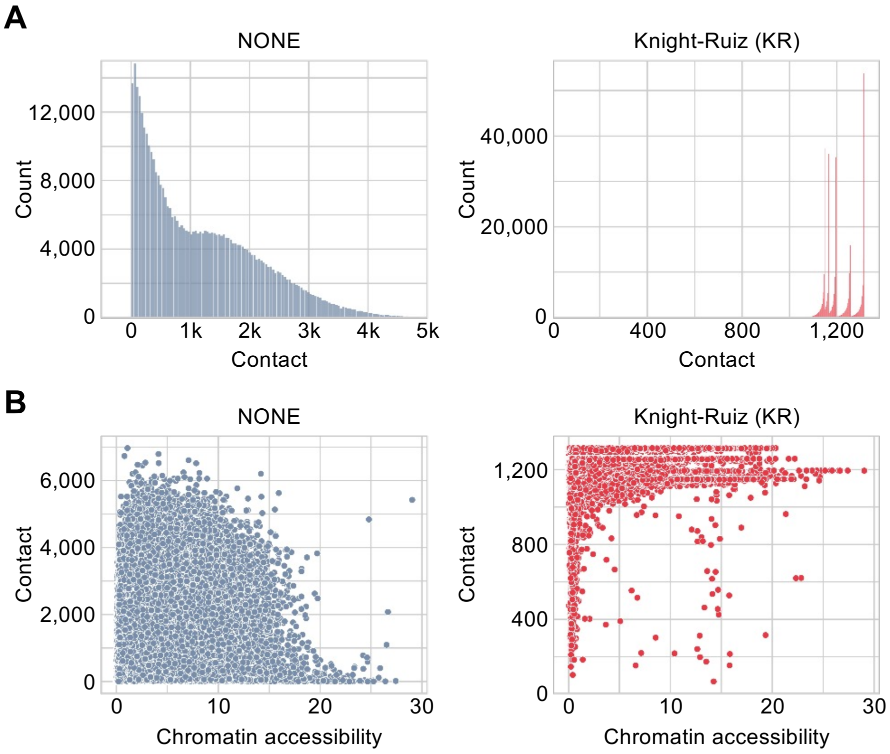

Supplementary Figure S17. Association between restriction enzyme efficiency and chromatin accessibility.

(**A**) Distribution of the sum of chromatin contact values for each single-restriction fragment. The distribution of contact values for each fragment is shown before normalization (NONE, left) or after normalization (KR, right). (**B**) Scatter plots showing the relationship between chromatin accessibility and the sum of contact values for each single-restriction fragment. The sum of contact values for each fragment is shown on the *y*-axis before normalization (NONE, left) or after normalization (KR, right). The *x*-axis indicates the chromatin accessibility level for each single-restriction fragment.

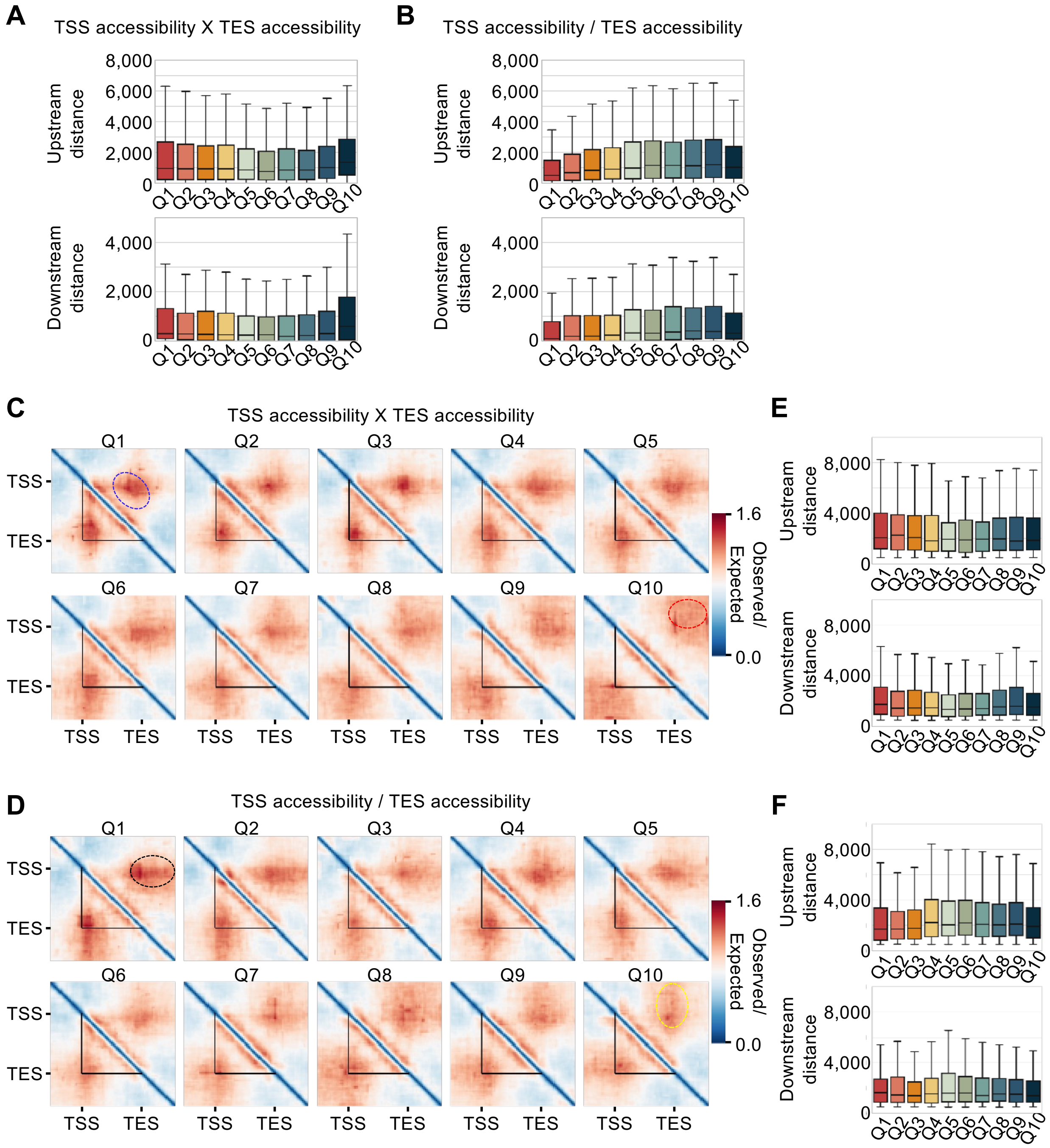

Supplementary Figure S18. Relationship between gene-to-gene linear distance and single-gene domain formation.

(**A**, **B**) Linear distance to the closest gene located at upstream (upper panels) and downstream (lower panels) regions. Genes longer than 2 kb were clustered into 10 quantiles according to the values of TSS accessibility × TES accessibility (**A**) or TSS accessibility / TES accessibility (**B**). (**C**, **D**) Domain structures of genes clustered by the values of TSS accessibility × TES accessibility (**C**) or TSS accessibility / TES accessibility (**D**). (**E**, **F**) Linear distance to the closest gene located at upstream (upper panels) and downstream (lower panels) regions. In (**C**−**F**), genes were filtered with 500 bp < linear distance cutoff < 10 kb. Filtered genes longer than 2 kb were clustered into 10 quantiles according to the TSS accessibility × TES accessibility values (**C**, **E**) or TSS accessibility / TES accessibility (**D**, **F**). In (**C**), a blue circle indicates a TSS–TES contact site, whereas a red circle indicates S-C subregion. In (**D**), a black circle indicates extended contacts between TSS and TES-downstream sequence, whereas a yellow circle indicates extended contacts between TSS-upstream sequence and TES. In (**A**), (**B**), (**E**) and (**F**), the box represents the interquartile range of the data, and the horizontal line indicates the median value. The whiskers indicate 1.5 times the interquartile range. Outliers are not shown.

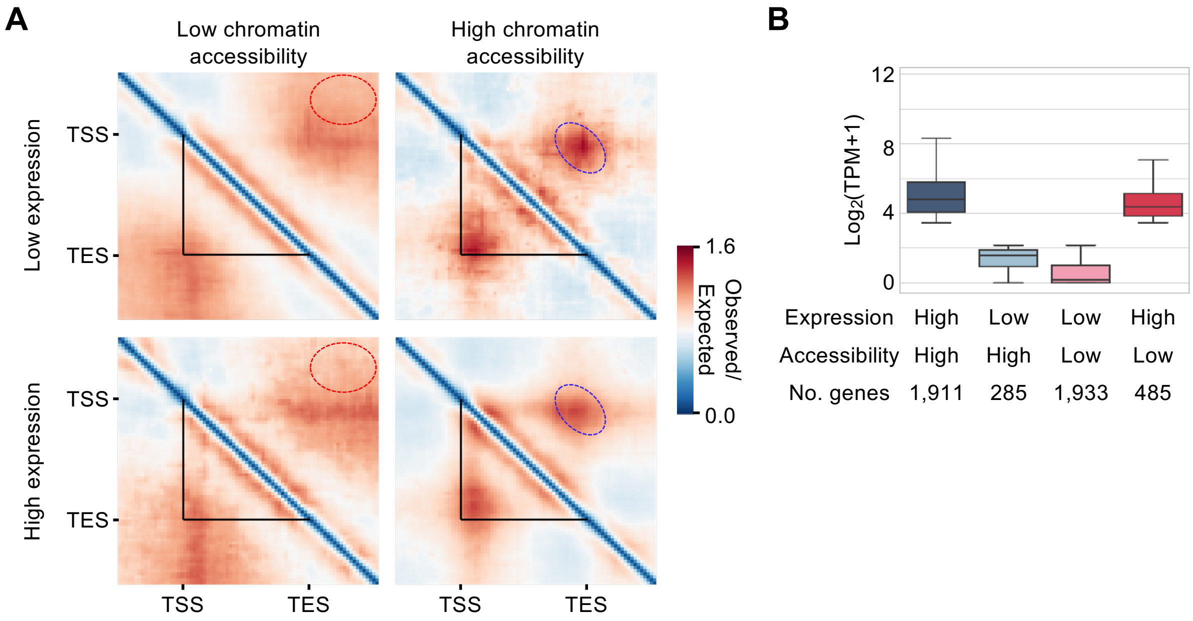

Supplementary Figure S19. Comparison on the impact of chromatin accessibility and gene expression in gene domain formation.

(**A**) Pile-up images of Hi-C contact matrices for *Arabidopsis* genes grouped based on their expression and chromatin accessibility levels. Black triangles indicate gene boundaries. Red circles indicate S-C subregion, whereas blue circles indicate a TSS–TES contact site. (**B**) Gene expression levels for each gene group. In (**A**) and (**B**), the gene expression clusters (Figure 2A) and the chromatin accessibility quantiles (Figure 3F) were employed for the analysis. The selected intersection between rarely expressed genes (C0-C1 of gene expression clusters)/highly expressed genes (C3-C4 of gene expression clusters) and rarely accessible genes (Q9-Q10 of TSS accessibility × TES accessibility quantiles)/highly accessible genes (Q1-Q2 of TSS accessibility × TES accessibility quantiles) were used for comparison. In (**B**), the box represents the interquartile range of the data, and the horizontal line indicates the median value. The whiskers indicate 1.5 times the interquartile range. Outliers are not shown.

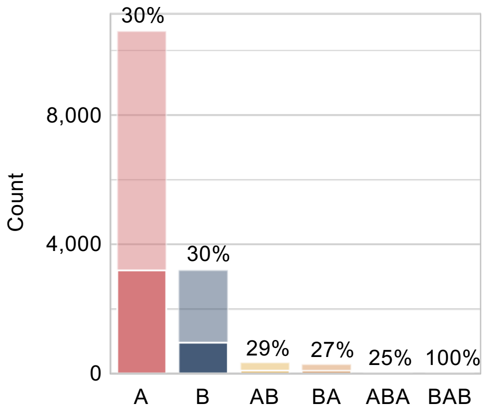

Supplementary Figure S20. Association between single-gene domain and the large A/B compartments.

Number of genes with strong domain structures in each large A/B compartment defined at 50-restriction-fragment resolution were shown. Genes longer than 2 kb were clustered into 10 quantiles according to the values of TSS accessibility × TES accessibility. Genes within Q1-Q3 quantiles were annotated as genes with strong domain structures. Boxes with light colors indicate the total number of *Arabidopsis* genes (longer than 2 kb) contained in each large A/B compartment. The ratio of the number of genes with strong gene domains over total number of genes in each A/B compartment is provided.

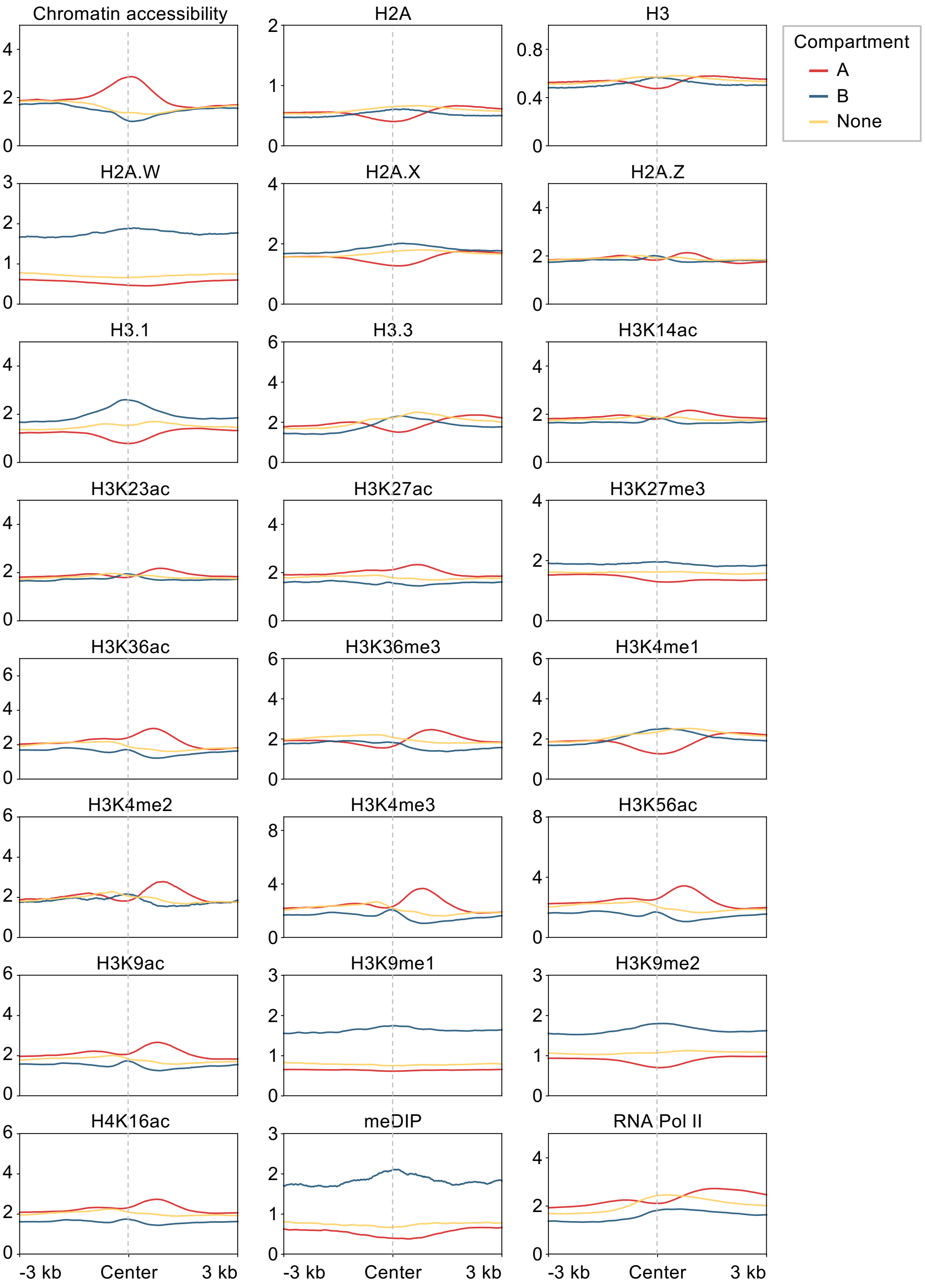

Supplementary Figure S21. Enrichment of various chromatin features in local A/B compartments. Metaplots for 24 chromatin features in each local A/B compartment were shown. Each 5-restriction-fragment bin in genic regions was annotated as A, B or none based on its first eigenvector value. The local A/B compartment was defined at 5-restriction-fragment resolution.

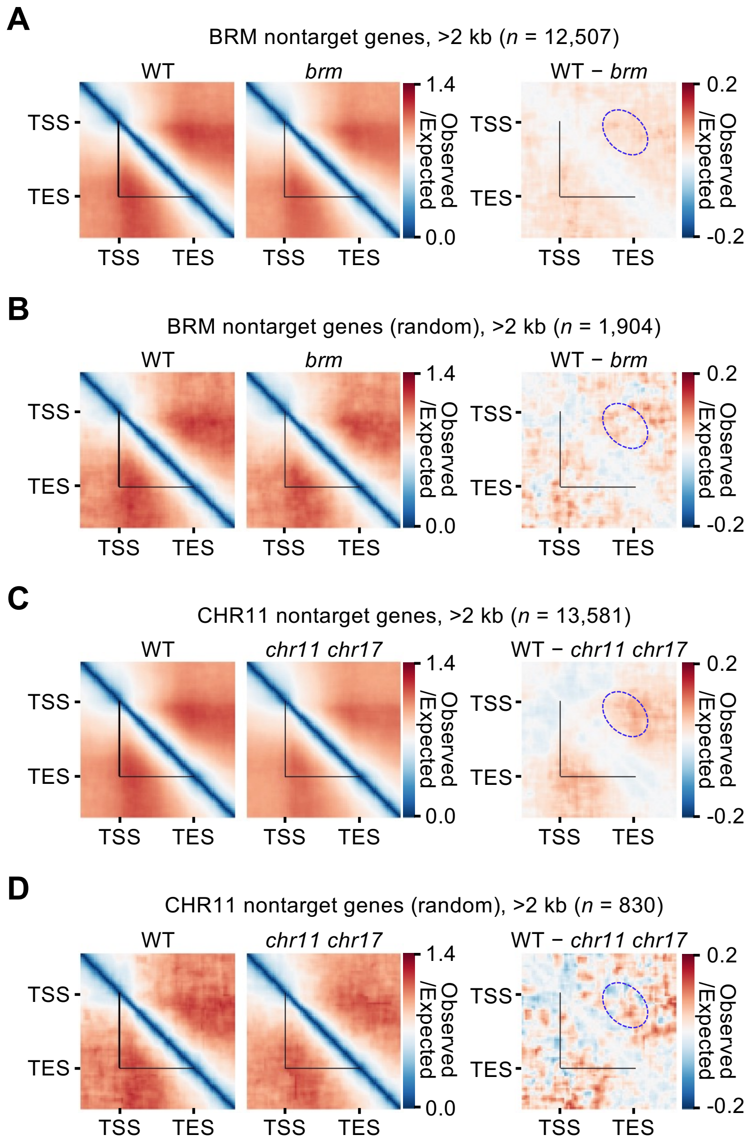

Supplementary Figure S22. Comparison of gene domain structures between wild type and chromatin remodeler mutants in *Arabidopsis*.

(**A–D**) Pile-up images of Hi-C contact matrices of nontarget genes of the chromatin remodeler proteins BRM (**A**, **B**) and CHR11 (**C**, **D**). In (**A**) and (**C**), all genes not targeted by each chromatin remodeler were used in the analysis. In (**B**) and (**D**), randomly selected nontarget genes with the same number of target genes were used for the analysis (Supplementary Table S4). In (**A–D**), left, wild-type Hi-C contact map; middle, Hi-C contact map of chromatin remodeler mutant; right, difference in Hi-C contact maps between the wild type and the chromatin remodeler mutant. Black triangles indicate gene boundaries. Blue circles indicate a TSS–TES contact site.

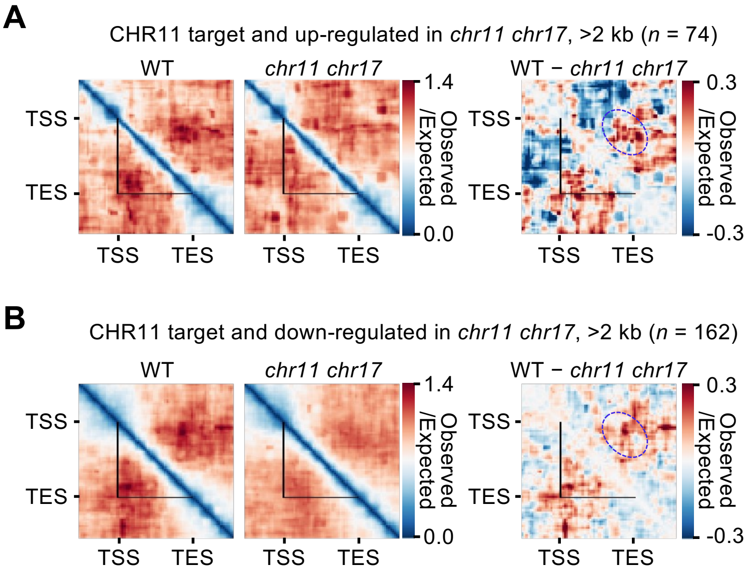

Supplementary Figure S23. Comparison of gene domain structures of CHR11-target genes between up-regulated and down-regulated in *chr11 chr17* mutant.

(**A**, **B**) Pile-up images of Hi-C contact matrices of CHR11-target genes up-regulated in *chr11 chr17* (**A**) or down-regulated in *chr11 chr17* (**B**). Left, wild-type Hi-C contact map; middle, Hi-C contact map of *chr11 chr17* mutant; right, difference in Hi-C contact maps between the wild type and the chromatin remodeler mutant. Black triangles indicate gene boundaries. Blue circles indicate a TSS–TES contact sites.

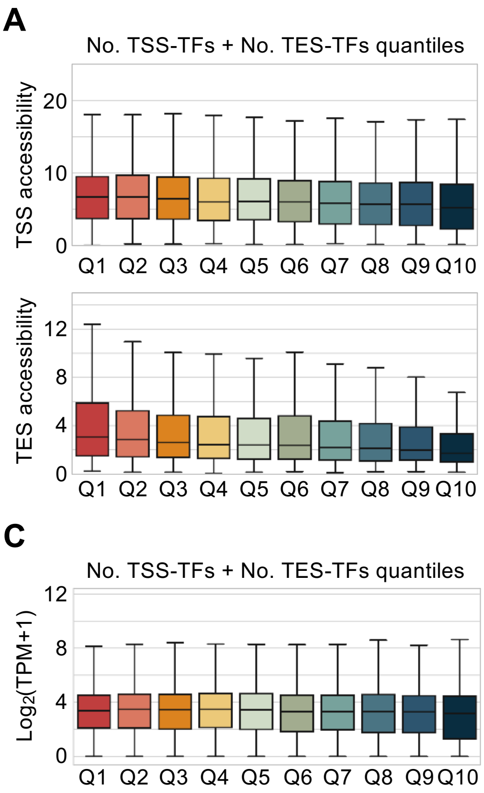

Supplementary Figure S24. Association between the transcription factor binding frequency and chromatin accessibility or gene expression.

(**A**, **B**) Chromatin accessibility levels in each quantile divided by the sum of the numbers of transcription factors binding to the TSS and TES (**A**) or by the difference between the number of transcription factors binding to the TSS and TES (**B**) of individual genes. Genes longer than 2 kb were used for the analysis. (**C**) Gene expression levels in each quantile divided by the sum of the numbers of transcription factors binding to the TSS and TES of individual genes. In (**A−C**), the box represents the interquartile range of the data, and the horizontal line indicates the median value. The whiskers indicate 1.5 times the interquartile range. Outliers are not shown.

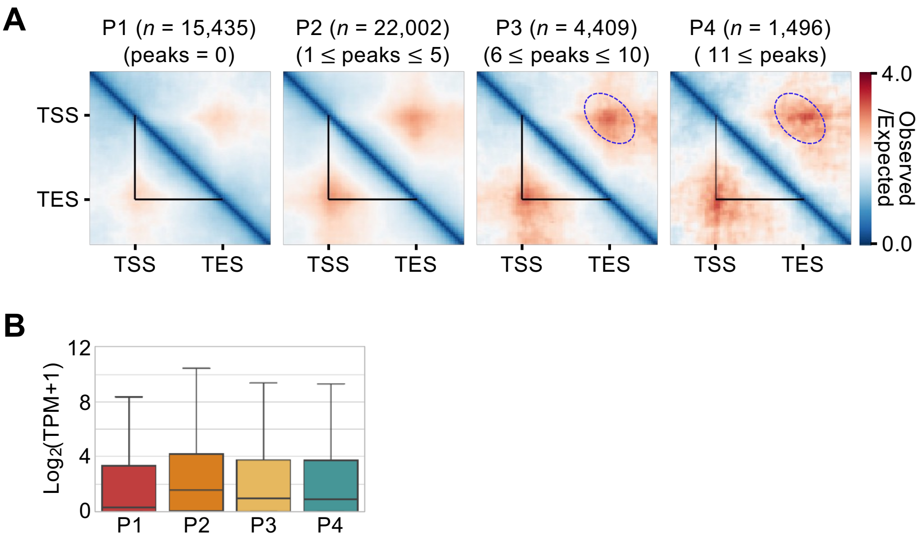

Supplementary Figure S25. Association between transcription factor binding frequency and single-gene domain formation in maize.

(**A**) Pile-up images of Hi-C contact matrices of maize genes clustered by the sum of the numbers of transcription factors binding to the TSS and TES. Black triangles indicate gene boundaries. Blue circles indicate a TSS–TES contact site. (**B**) Gene expression levels of each cluster shown in (**A**). The box represents the interquartile range of the data, and the horizontal line indicates the median value. The whiskers indicate 1.5 times the interquartile range. Outliers are not shown. In (**A**) and (**B**), genes longer than 2 kb in maize were divided into 4 clusters based on the numbers of transcription factors binding to the TSS and TES.

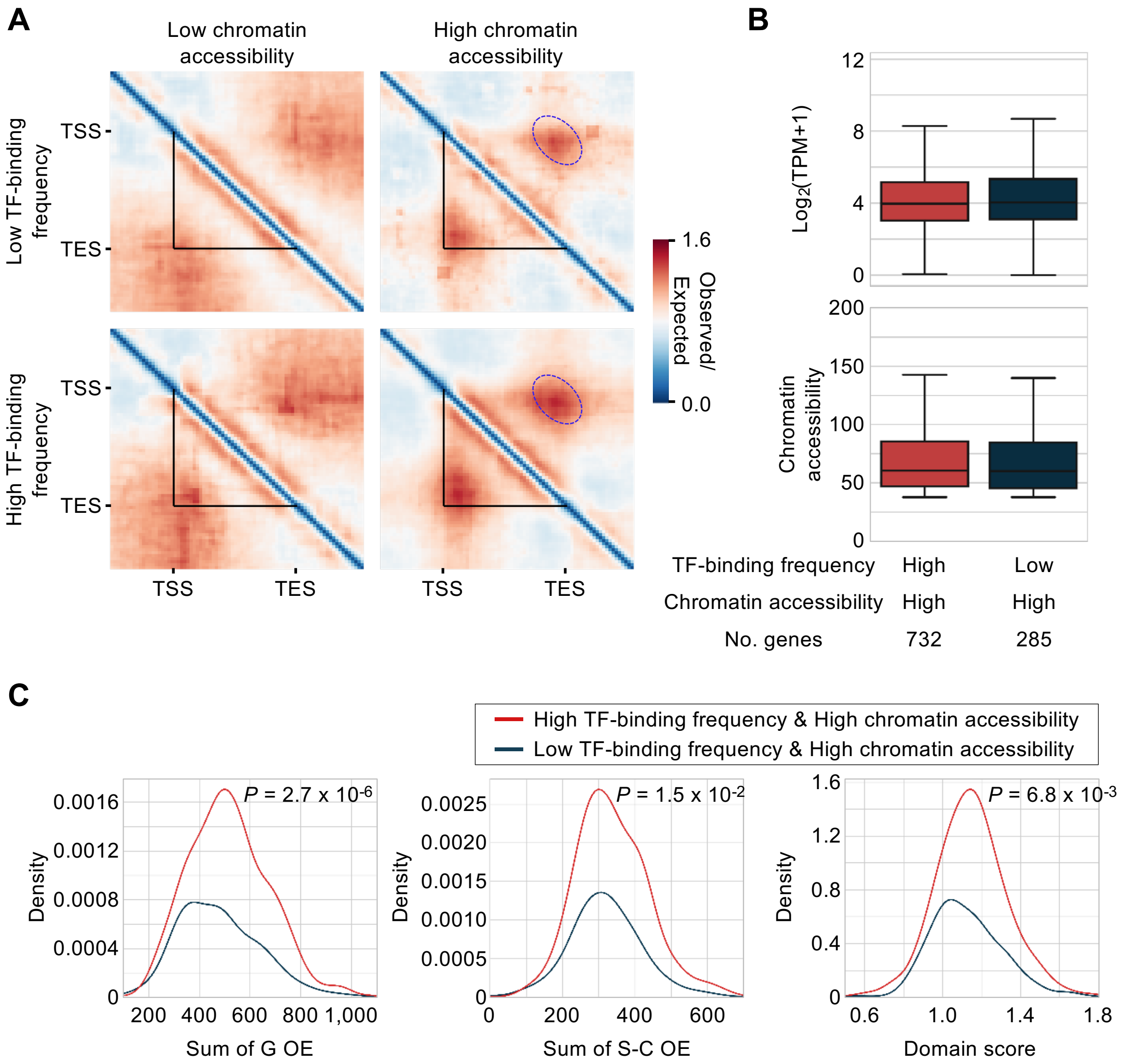

Supplementary Figure S26. Impact of transcription factor binding to gene domain formation. (**A**) Pile-up images of Hi-C contact matrices for *Arabidopsis* genes grouped by transcription factor binding frequency and chromatin accessibility. Black triangles indicate gene boundaries. Blue circles indicate a TSS–TES contact sites. (**B**) Gene expression levels (upper panels) and the TSS accessibility × TES accessibility values (lower panels) for each group of genes. The box represents the interquartile range of the data, and the horizontal line indicates the median value. The whiskers indicate 1.5 times the interquartile range. Outliers are not shown. (**C**) Kernel density plots of the sum of OE values at G and S-C subregions and domain score (DS). The *P* value was calculated by a two-sided Mann–Whitney test. In (**A-C**), the transcription factor binding frequency quantiles (Figure 4B) and the chromatin accessibility quantiles (Figure 3F) were employed. The selected intersection between genes with high transcription factor binding frequency (Q1-Q2 of the sum of the numbers of transcription factors binding to the TSS and TES of individual genes)/low transcription factor binding frequency (Q9-Q10 of the sum of the numbers of transcription factors binding to the TSS and TES of individual genes) and rarely accessible genes (Q9-Q10 of TSS accessibility × TES accessibility quantiles)/highly accessible genes (Q1-Q2 of TSS accessibility × TES accessibility quantiles) were used for comparison.

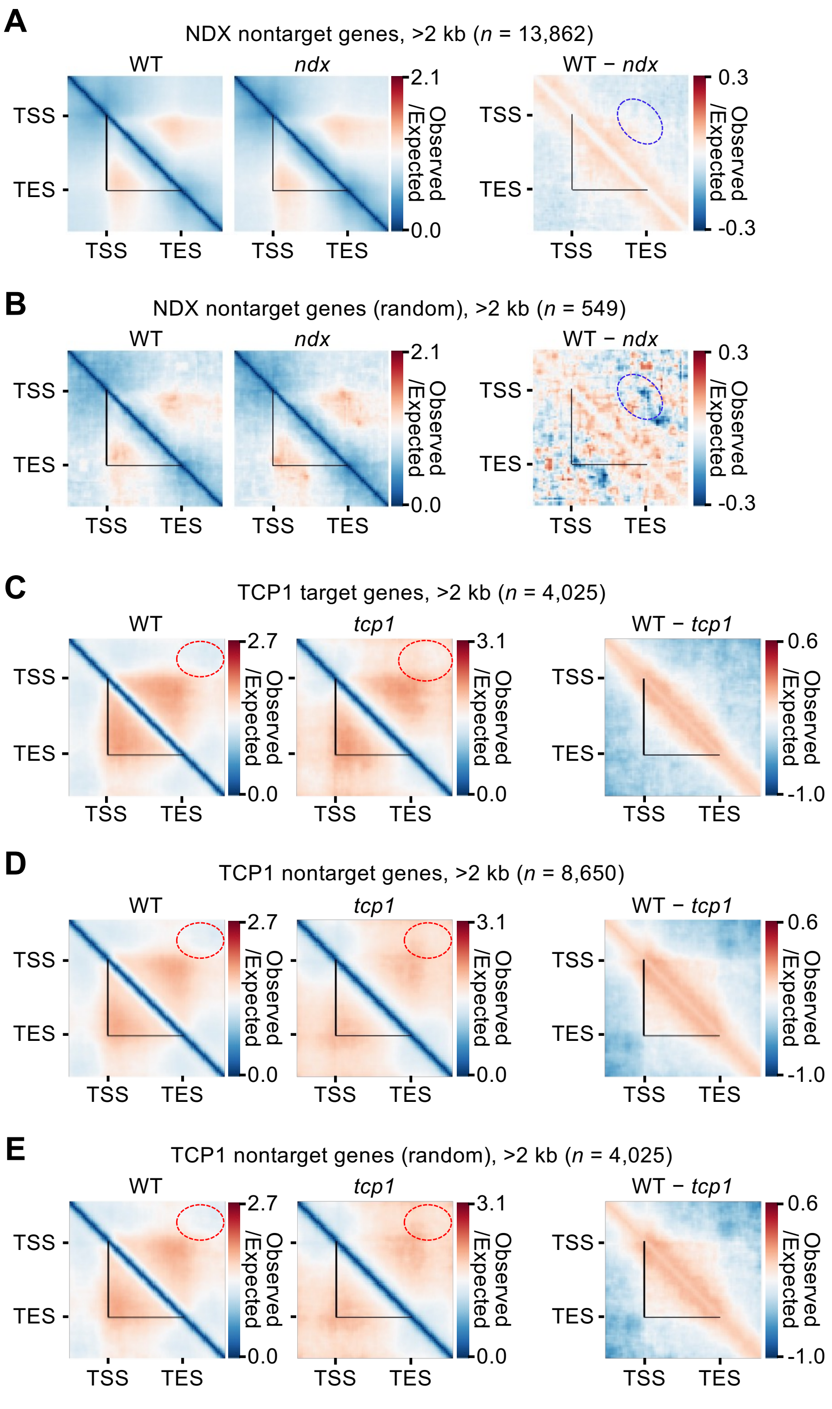

Supplementary Figure S27. Comparison of gene domain structures between wild type and transcription factor mutants in *Arabidopsis* and *M. polymorpha*.

(**A, B**) Pile-up images of Hi-C contact matrices of nontarget genes of NDX. (**C**) Pile-up images of Hi-C contact matrices of TCP1-target genes. (**D**, **E**) Pile-up images of Hi-C contact matrices of nontarget genes of TCP1. In (**A**) and (**D**), all genes not targeted by each transcription factor were used in the analysis. In (**B**) and (**E**), randomly selected nontarget genes with the same number of target genes were used for the analysis (Supplementary Table S5). In (**A–E**), left, wild-type Hi-C contact map; middle, Hi-C contact map of transcription factor mutant; right, difference in Hi-C contact maps between the wild type and the transcription factor mutant. Black triangles indicate gene boundaries. Red circles indicate S-C subregion, whereas blue circles indicate a TSS–TES contact site.

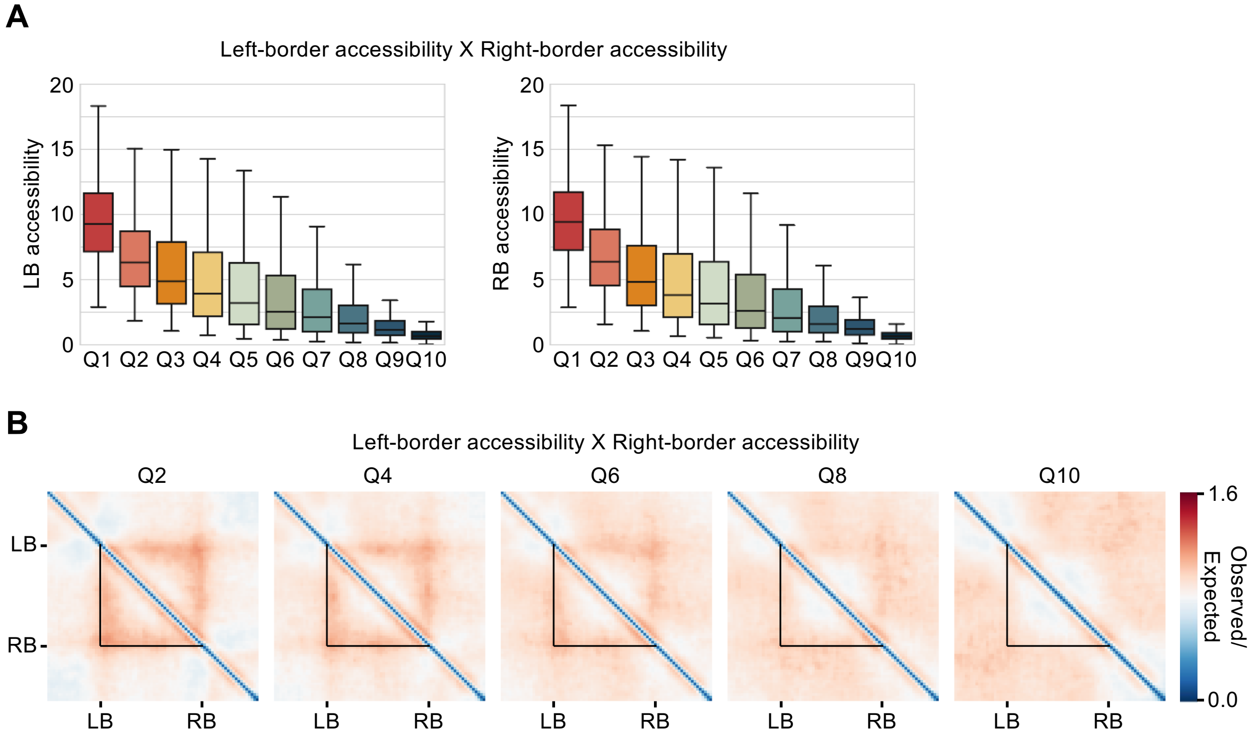

Supplementary Figure S28. Dual-gene domains in *Arabidopsis*.
(**A**) Chromatin accessibility levels at gene domain boundaries. Gene pairs were divided into 10 quantiles according to the values of left-border (LB) accessibility (TES or TSS) × right-border (RB) accessibility (TES or TSS). Accessibility values at the left borders (left panel) and right borders (right panel) of dual-gene domains in each quantile are shown. The box represents the interquartile range of the data, and the horizontal line indicates the median value. The whiskers indicate 1.5 times the interquartile range. Outliers are not shown. (**B**) Pile-up images of Hi-C contact matrices of the dual-gene domain quantiles Q2, Q4, Q6, Q8, and Q10. Black triangles indicate dual-gene domain boundaries.

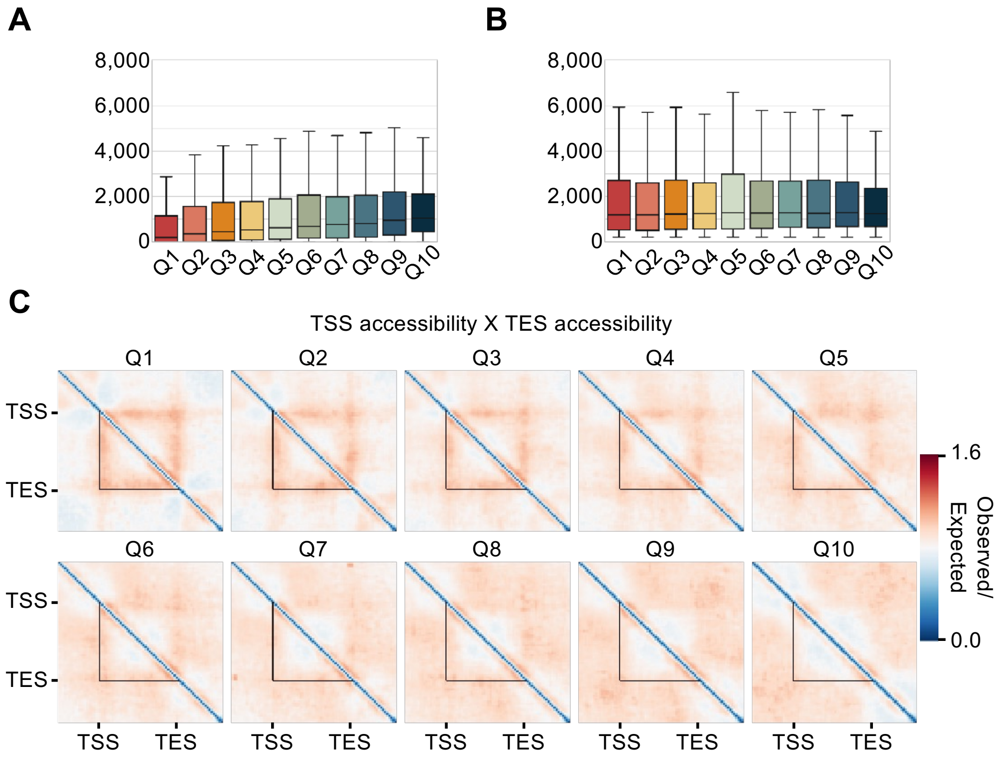

Supplementary Figure S29. Association between gene-to-gene linear distance and dual-gene domain formation.

(**A**, **B**) Linear distance between neighboring genes in each dual-gene domain quantile. The gene pairs located within 20 kb were divided into 10 quantiles according to the values of left-border (LB) accessibility × right-border (RB) accessibility. In (**B**), gene pairs were filtered with the linear distance threshold of > 200 bp and then divided into 10 quantiles according to the values of LB accessibility × RB accessibility. In (**A**) and (**B**), the box represents the interquartile range of the data, and the horizontal line indicates the median value. The whiskers indicate 1.5 times the interquartile range. Outliers are not shown. (**C**) Dual-gene domain structures of gene pairs clustered by the values of LB accessibility × RB accessibility with the linear distance threshold of > 200 bp. Black triangles indicate dual-gene domain boundaries.

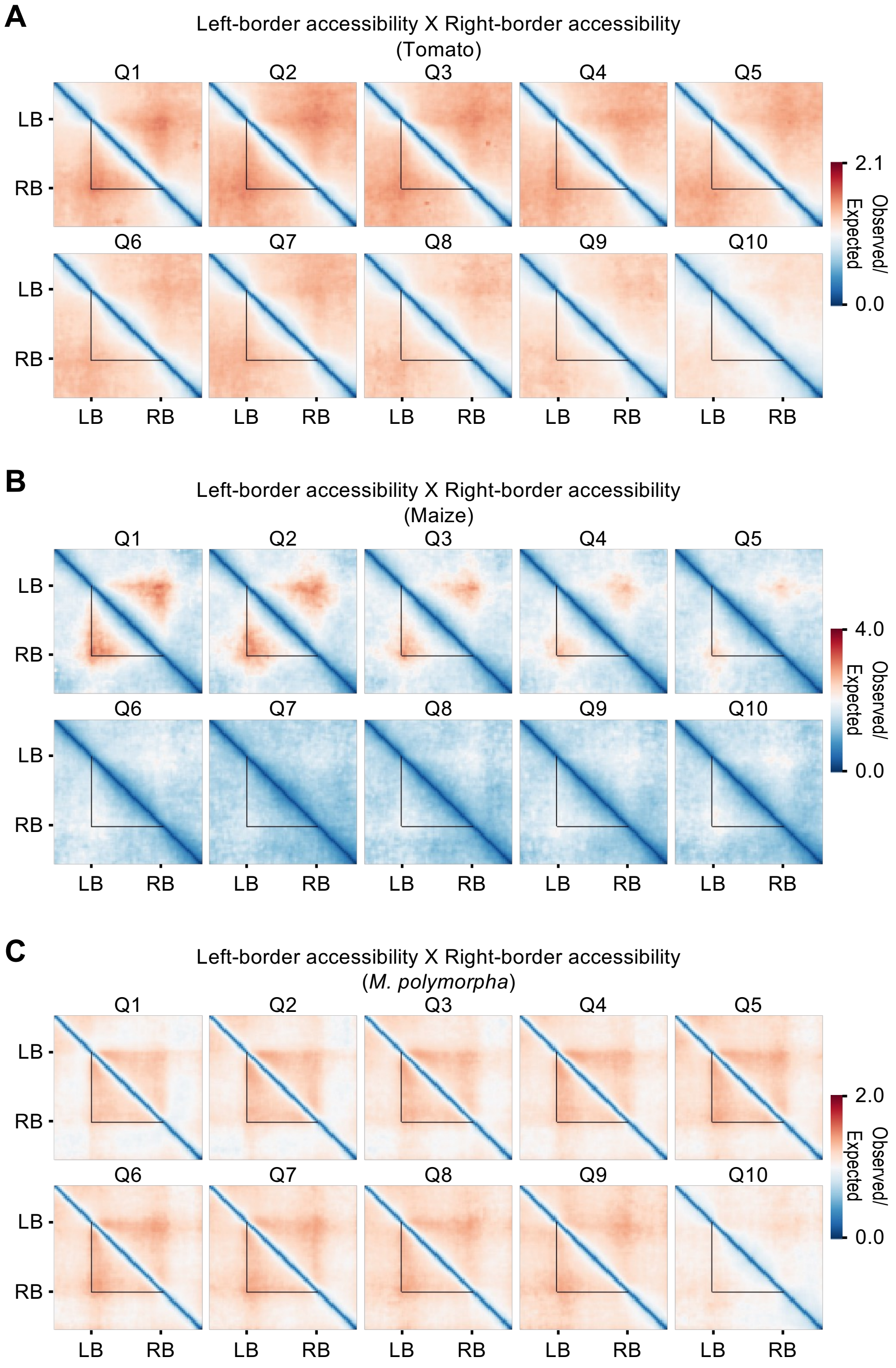

Supplementary Figure S30. Dual-gene domains in several plant species.

(**A**-**C**) Pile-up images of Hi-C contact matrices of tomato (**A**), maize (**B**) and *M. polymorpha* (**C**) dual-gene domains. Gene pairs located within 20 kb were divided into 10 quantiles according to the values of left-border (LB) accessibility × right-border (RB) accessibility. The Hi-C contact matrices at two-restriction-fragment resolution were used for the analysis. Black triangles indicate dual-gene domain boundaries.

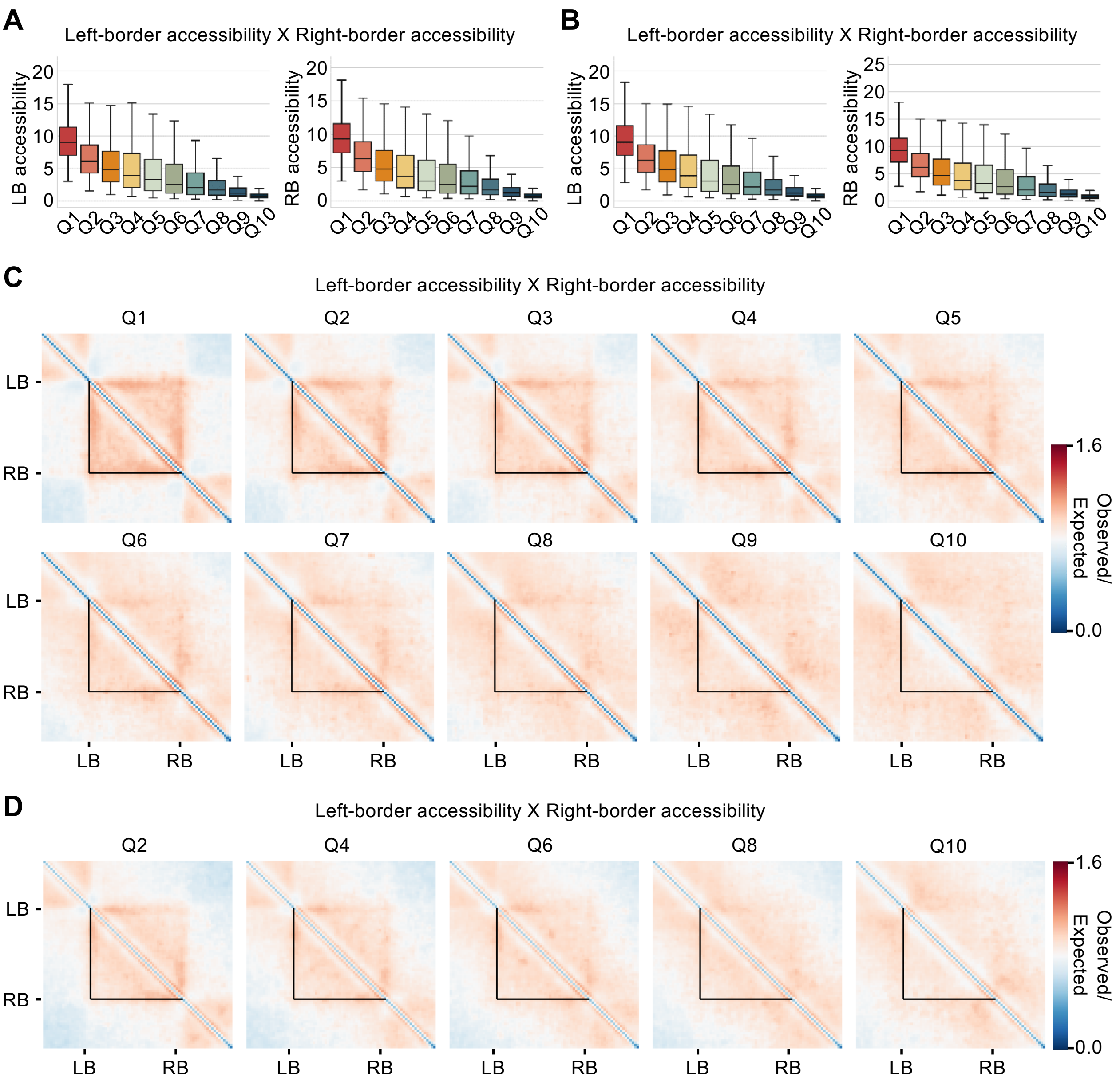

Supplementary Figure S31. Multigene domains in *Arabidopsis*.

(**A**, **B**) Chromatin accessibility in each quantile. Triple-gene domains (**A**) and quadruple-gene domains (**B**) were divided into 10 quantiles according to the values of left-border (LB) accessibility × right-border (RB) accessibility. Accessibility values at the left borders (left panel) and right borders (right panel) of genes in each quantile are shown. The box represents the interquartile range of the data, and the horizontal line indicates the median value. The whiskers indicate 1.5 times the interquartile range. Outliers are not shown. (**C**, **D**) Pile-up images of Hi-C contact matrices of triple-gene domains (**C**) and quadruple-gene domains (**D**). Black triangles indicate multigene domain boundaries. In (**D**), quadruple-gene domain structures of Q2, Q4, Q6, Q8, and Q10 are shown.

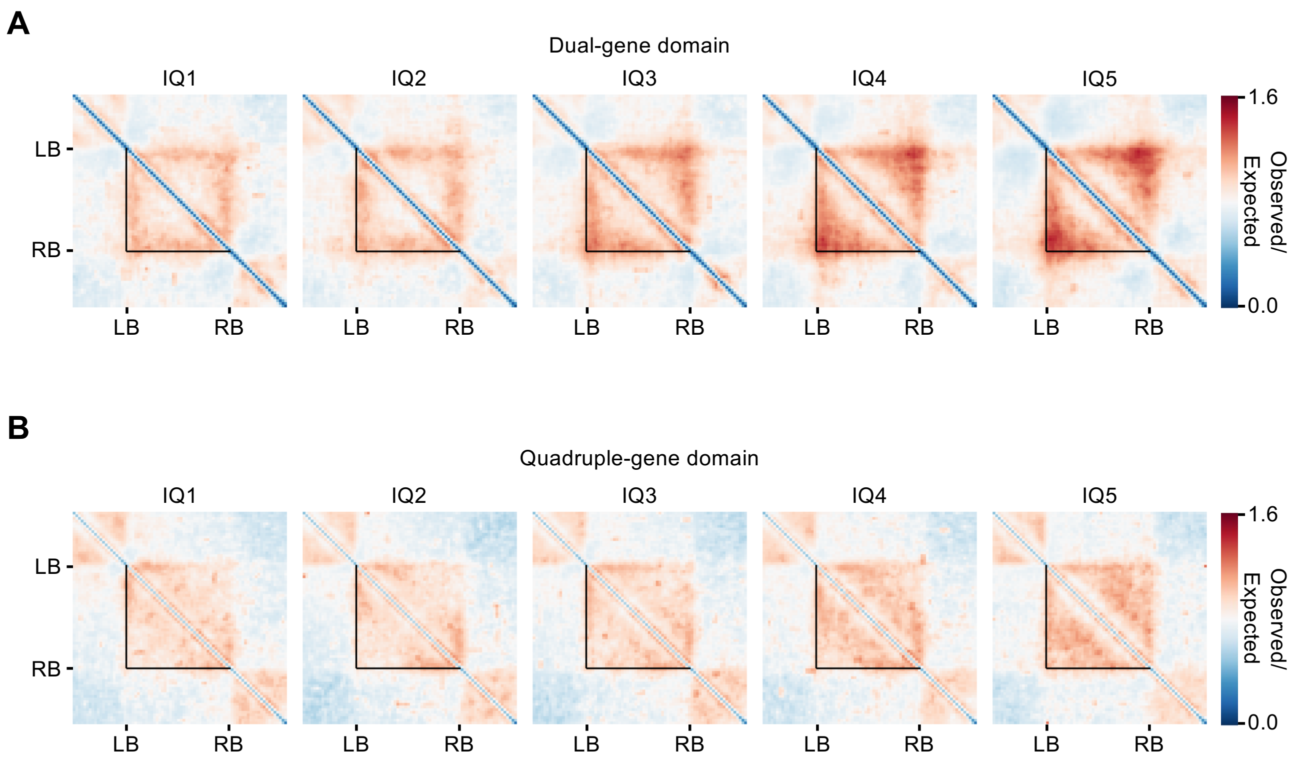

Supplementary Figure S32. Effect of internal accessibility on multigene domain formation.
(**A**, **B**) Pile-up images of Hi-C contact matrices of dual-gene domains (**A**) and quadruple-gene domains (**B**) divided according to internal gene-border accessibility (internal accessibility). Dual-gene domains (**A**) and quadruple-gene domains (**B**) were first divided into 10 quantiles according to the values of left-border (LB) accessibility × right-border (RB) accessibility. Then, the Q1 quantile was further divided into five sub-quantiles (IQ1–IQ5) according to the multiplied values of internal accessibility. Internal accessibility levels decrease from left to right (IQ1 > IQ2 > IQ3 > IQ4 > IQ5). Black triangles indicate multigene domain boundaries.

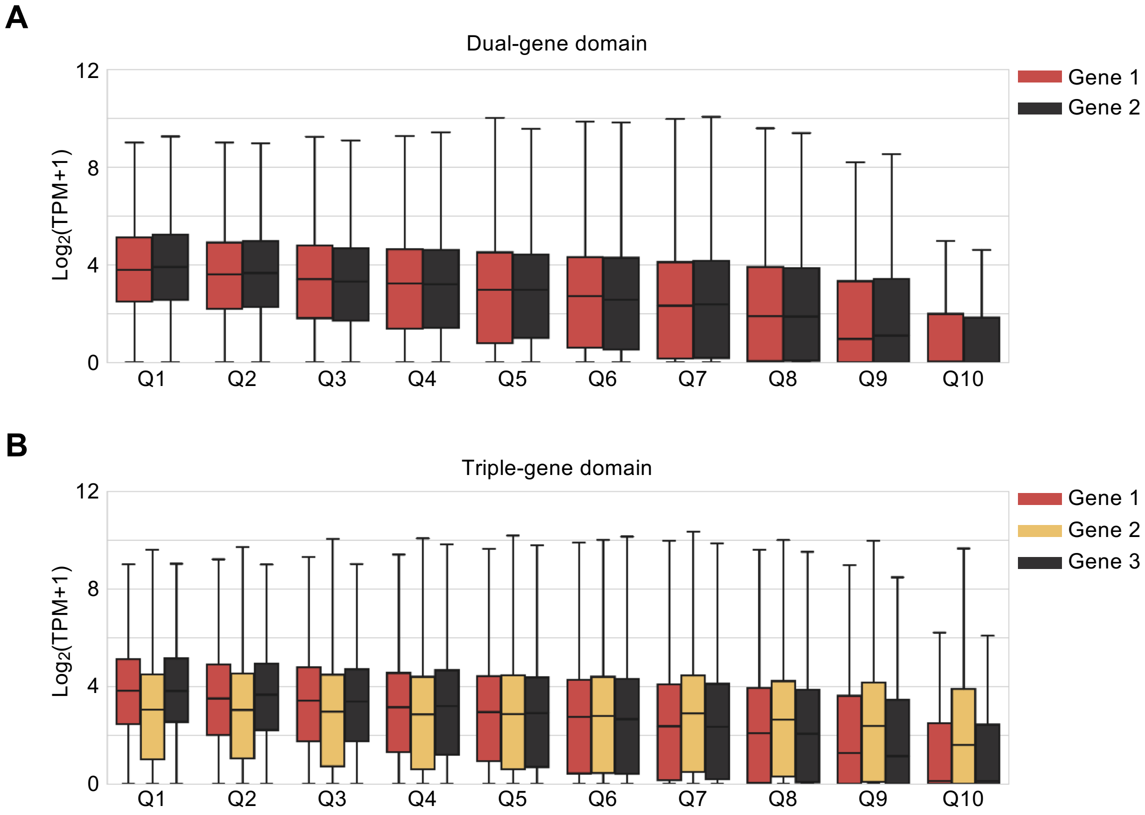

Supplementary Figure S33. Expression levels of constituent genes of multigene domains.
(**A**, **B**) Expression levels of constituent genes of dual-gene domains (**A**) and triple-gene domains (**B**). Domains were divided into 10 quantiles according to the values of left-border accessibility × right-border accessibility. The *x*-axis indicates the multiple quantiles of dual-gene domains (**A**) and triple-gene domains (**B**). The *y*-axis indicates expression levels shown as Log_2(TPM+1). The box represents the interquartile range of the data, and the horizontal line indicates the median value. The whiskers indicate 1.5 times the interquartile range. Outliers are not shown. Gene 1 indicates the gene located at the left border of multigene domain.

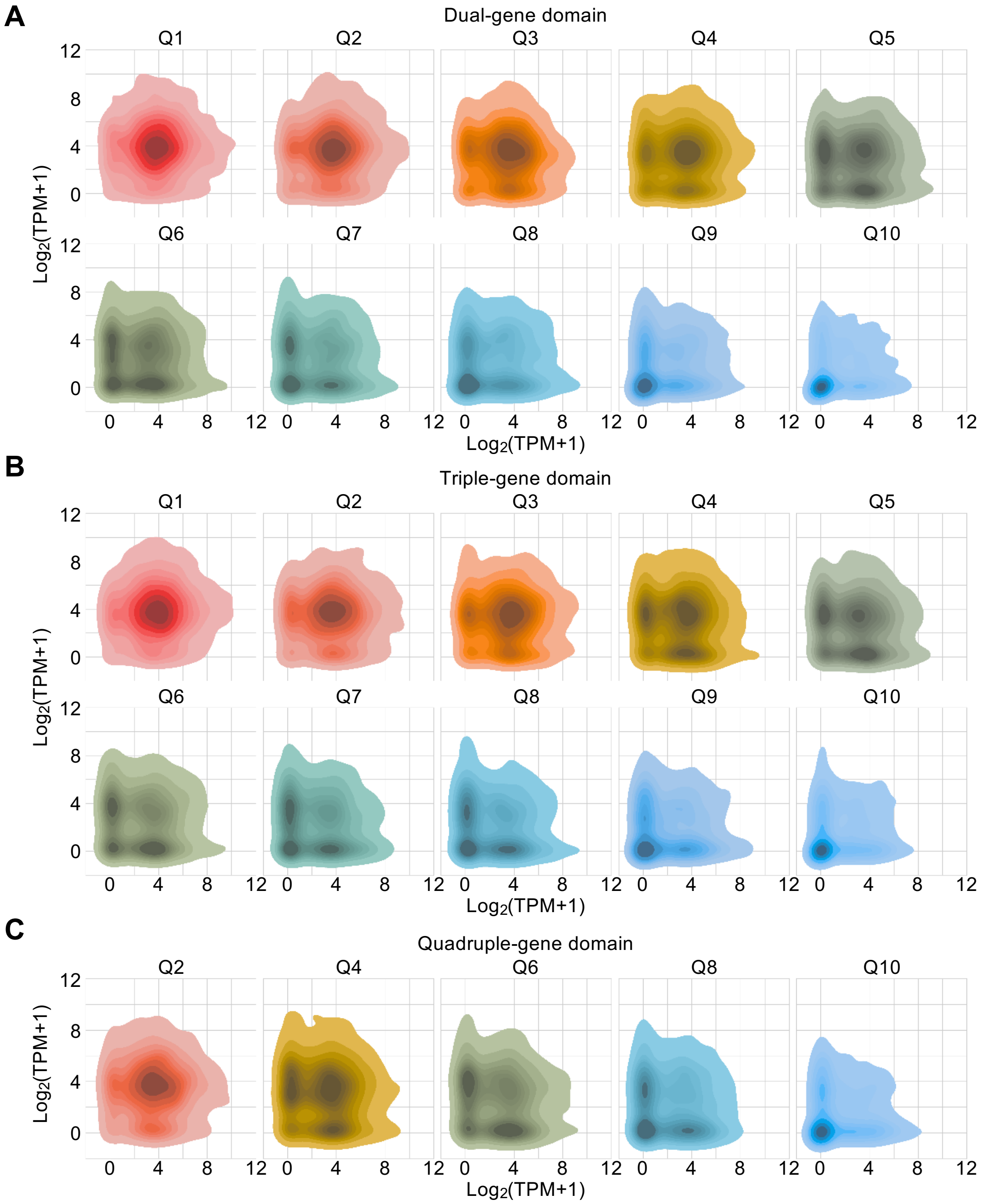

Supplementary Figure S34. Co-expression of gene pairs located at boundary regions of multigene domains.

(**A–C**) Two-dimensional kernel density plots of the expression levels of gene pairs located at multigene domain boundaries. Gene domains were divided into 10 quantiles according to the values of left-border accessibility × right-border accessibility. Expression density of boundary gene pairs in dual-gene domains (**A**), triple-gene domains (**B**), and quadruple-gene domains (**C**) is shown. The *x*-axis indicates expression levels of genes located at the left border of multigene domains, and the *y*-axis indicates expression levels of genes located at the right border of multigene domains. Log_2(TPM+1) values were used for the analysis.

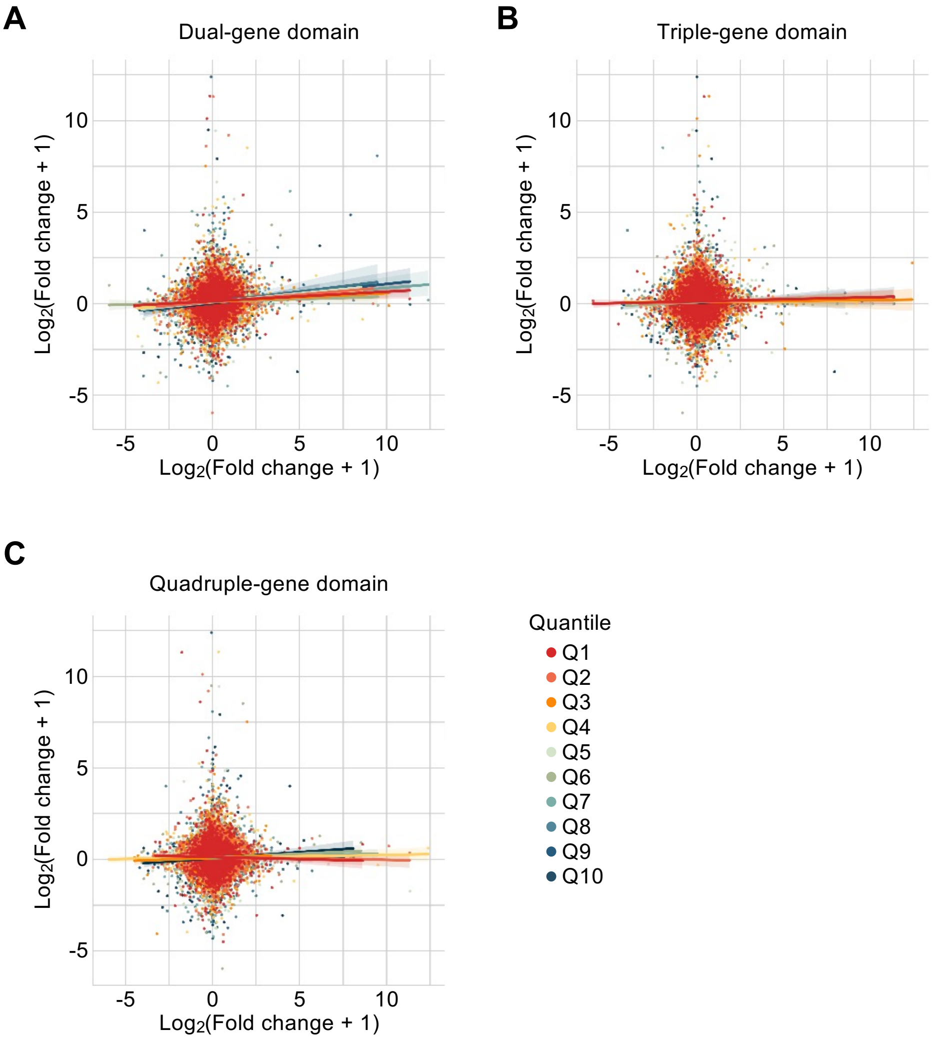

Supplementary Figure S35. Co-regulation of gene pairs located within multigene domain boundaries. (**A**–**C**) Expression changes of genes located at domain boundaries in response to heat. Gene domains were divided into 10 quantiles according to the values of left-border accessibility × right-border accessibility. Expression changes in boundary genes of dual-gene domains (**A**), triple-gene domains (**B**), and quadruple-gene domains (**C**) are shown. The *x*-axis indicates Log_2(fold change + 1) of genes located at the left boundary, and the *y*-axis indicates Log_2(fold change + 1) of genes located at the right boundary. Linear regression lines are indicated. Gene 1 indicates the gene located at the left border of multigene domain.

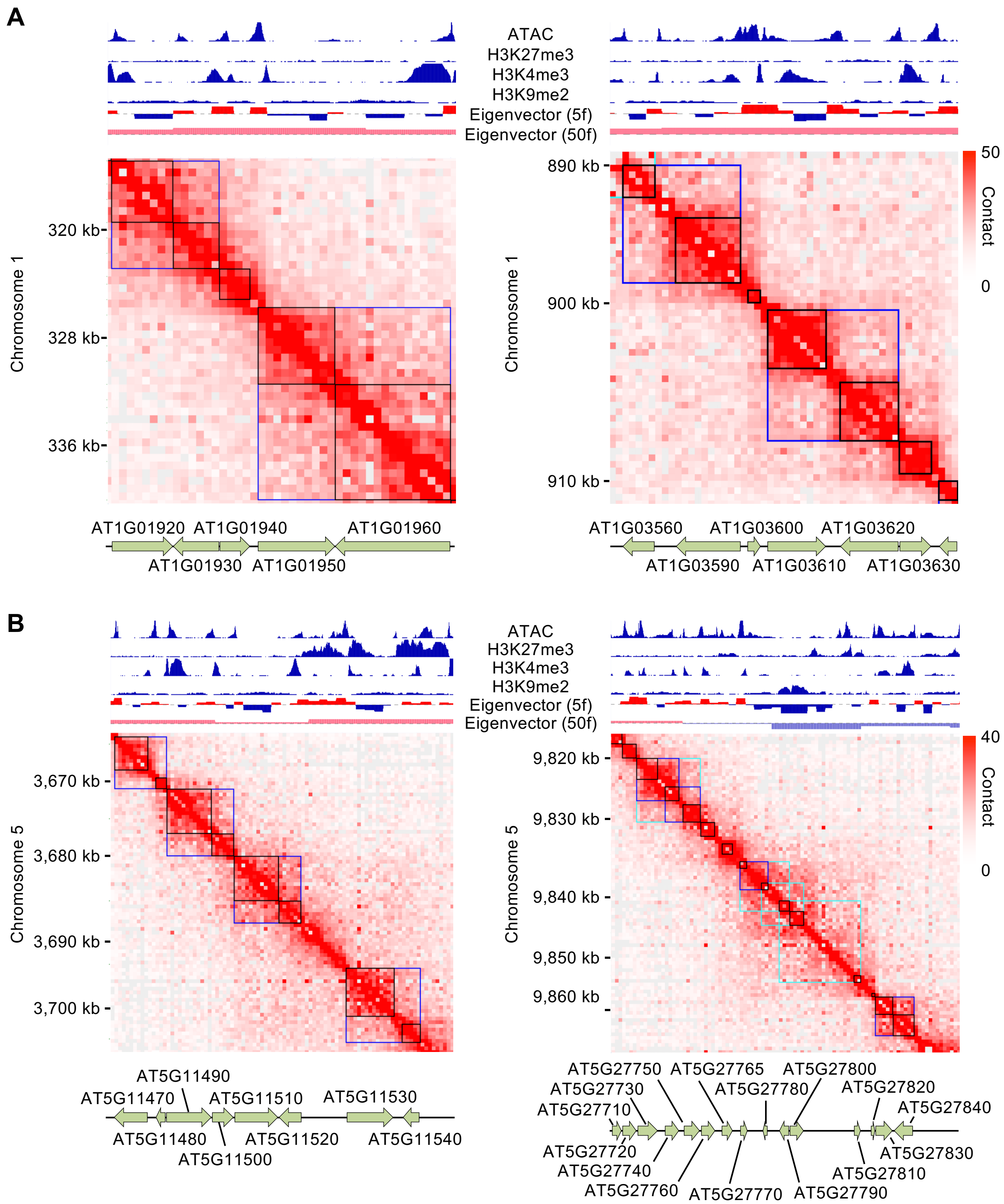

Supplementary Figure S36. Example regions of gene domains with various chromatin features.
(**A**, **B**) Several example regions with information on gene domains, chromatin accessibility (ATAC), H3K27me3, H3K4me3, H3K9me2, and A/B compartments at 5-restriction-fragment resolution and 50-restriction-fragment resolution are shown. Black boxes in the Hi-C contact maps indicate individual genes. Blue and sky blue boxes in the Hi-C contact maps indicate dual-gene domains (quantiles Q1–Q4; values of left-border accessibility × right-border accessibility / multiplied values of internal accessibility) and triple-gene domains (quantiles Q1–Q3; values of left-border accessibility × right-border accessibility / multiplied values of internal accessibility), respectively. Hi-C contact maps at two-restriction-fragment resolution are shown.

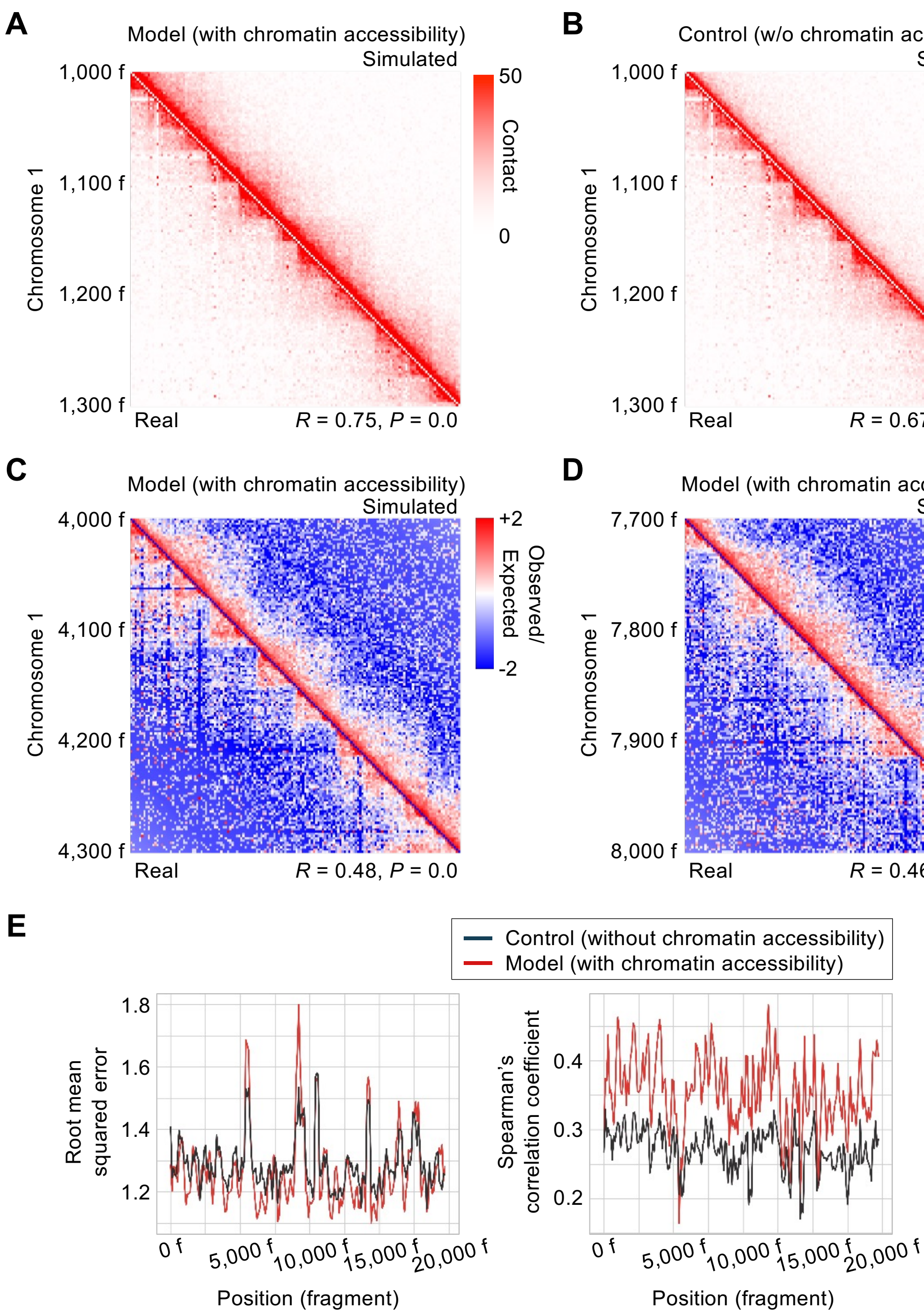

Supplementary Figure S37. Simulated Hi-C contact maps with chromatin accessibility data.
(**A**, **B**) Simulated Hi-C contact maps at two-restriction-fragment resolution with (**A**) or without (**B**) chromatin accessibility input. (**C**, **D**) Several example regions of simulated OE maps. In (**A**−**D**), the upper-right triangles display the simulated Hi-C maps, while the lower-left triangles show real Hi-C maps at two-restriction-fragments (2 f) resolution. The Spearman's correlation coefficient between the simulated Hi-C maps and the real Hi-C maps, along with the *P*-value, is indicated. Interacting bins (<50 fragments in gap distance between anchors) were used for the correlation coefficient calculation. (**E**) Comparison of root mean squared error and Spearman's correlation coefficient between simulated OE maps with and without chromatin accessibility information at broader regions. *Arabidopsis* chromosome 1: 1 – 20,000 f (1 – 5,296,408 bp) regions were used in the analysis. Simulation was performed for each unit of 300 restriction fragments.

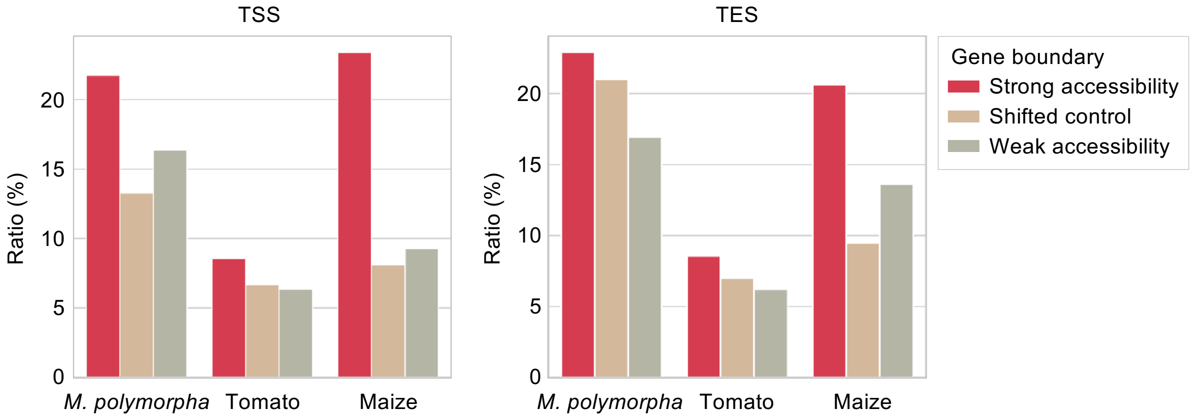

Supplementary Figure S38. Overlaps between conventional TAD boundaries and gene domain boundaries.

Bar plots represent the percentages of conventional TAD boundaries overlapping with gene domain boundaries in various plant species. Conventional TAD structures were defined at 5 kb resolution. Gene borders (TSSs and TESs) were clustered into 10 quantiles according to the chromatin accessibility level, and Q1(strong gene domain boundaries) and Q10 (weak gene domain boundaries) quantiles were used in the analysis. For shifted controls, 10 kb upstream regions of strong TSSs and 10 kb downstream regions of strong TESs were collected.
